# Anti-CD117 CAR T cells incorporating a safety switch eradicate human acute myeloid leukemia and hematopoietic stem cells

**DOI:** 10.1016/j.omto.2023.08.002

**Published:** 2023-08-20

**Authors:** Chiara F. Magnani, Renier Myburgh, Silvan Brunn, Morgane Chambovey, Marianna Ponzo, Laura Volta, Francesco Manfredi, Christian Pellegrino, Steve Pascolo, Csaba Miskey, Nicolás Sandoval-Villegas, Zoltán Ivics, Judith A. Shizuru, Dario Neri, Markus G. Manz

## Main text

(Molecular Therapy: Oncolytics *30*, 56–71; September 2023)

In the originally published version of this article, Nicolás Sandoval-Villegas was not present in the author list. He completed the western blot analysis for this study and should have been mentioned as an author.

The authors apologize for this error, and this has been corrected online.

