# Supplementary material for: Anti-CD117 CAR T cells incorporating a safety switch eradicate human acute myeloid leukemia and hematopoietic stem cells
Source: Mol Ther Oncolytics. 2023 Jul 19;30:56–71. doi: 10.1016/j.omto.2023.07.003 (PMC10424000; doi:10.1016/j.omto.2023.07.003)
Supplement: Document S1. Figures S1–S8 and Table S1 [file mmc1.pdf]

OMTO, Volume *30*

Supplemental information

Anti-CD117 CAR T cells incorporating a safety switch eradicate human acute myeloid leukemia and hematopoietic stem cells

Chiara F. Magnani, Renier Myburgh, Silvan Brunn, Morgane Chambovey, Marianna Ponzo, Laura Volta, Francesco Manfredi, Christian Pellegrino, Steve Pascolo, Csaba Miskey, Zoltán Ivics, Judith A. Shizuru, Dario Neri, and Markus G. Manz

Supplemental Information

Table S1. List of fluorescently labeled antibodies/proteins used for flow cytometry.

Target	Clone	Fluorochrome	Supplier
Protein-A	n/a	FITC	Thermo Fisher
CD3	OKT3	APC	BioLegend
CD4	RPA-T4	FITC	Invitrogen
CD8	SK1	PerCP-Cy5.5	BioLegend
CD45RA	HI100	Brilliant Violet 711™	BioLegend
CD62L	DREG-56	PB	BioLegend
CD117	104D2	PE-Cy7	Thermo Fisher
CD27	M-T271	BV510	Biolegend
CD95	DX2	BV786	BD Biosciences
CD279	EH12.2H7	APC Cy7	Biolegend
CD223	3DS223H	Alexa-Fluor 700	BD Biosciences
CD366	F38-2E2	PE	Ebiosciences
mouse CD45	30-F11	PerCP-Cy5.5	BioLegend
CD34	QBend-10	PE	Thermo Fisher
CD33	WM53	Brilliant Violet 711™	BioLegend
CD19	SJ21C1	FITC	BioLegend
CD45	HI30	eFluor™ 450	Thermo Fisher
HLA-A2	BB7.2	APC-780	BD Biosciences

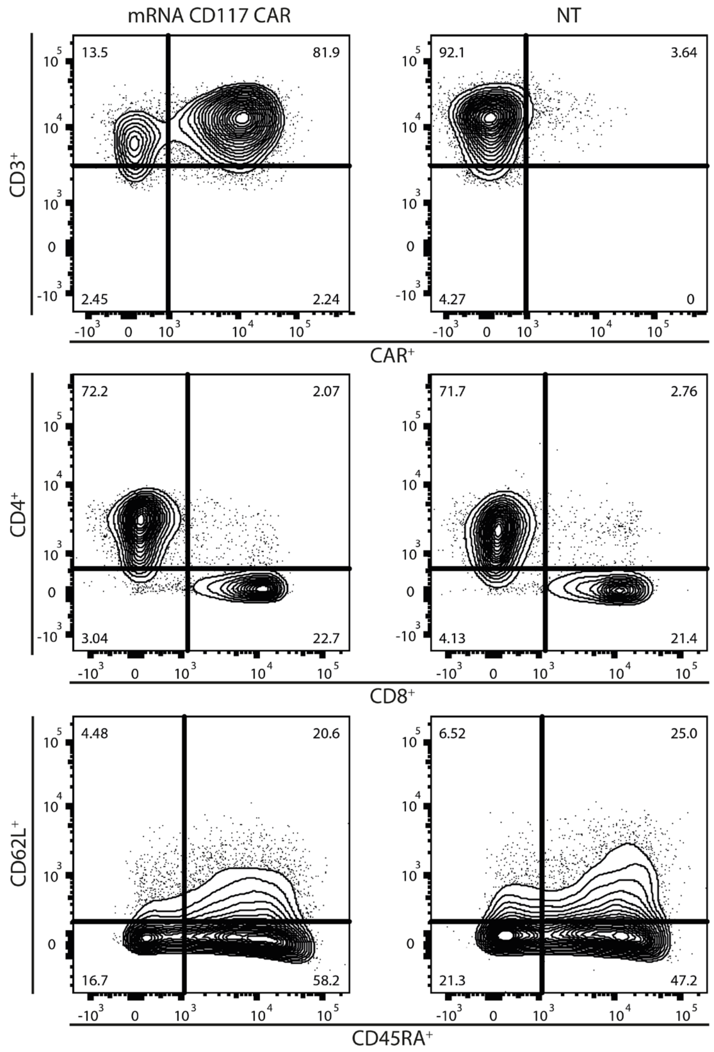

Figure S1: Phenotype of T cells electroporated with mRNA CD117CAR.
Purified T cells were stimulated and electroporated with 10 µg of CAR mRNA or in absence of DNA (not transfected, NT). Flow cytometric immunophenotyping by dual density plots in one representative donor (n = 5). CD3/CAR, CD4/CD8, CD45RA/CD62L expression were measures.

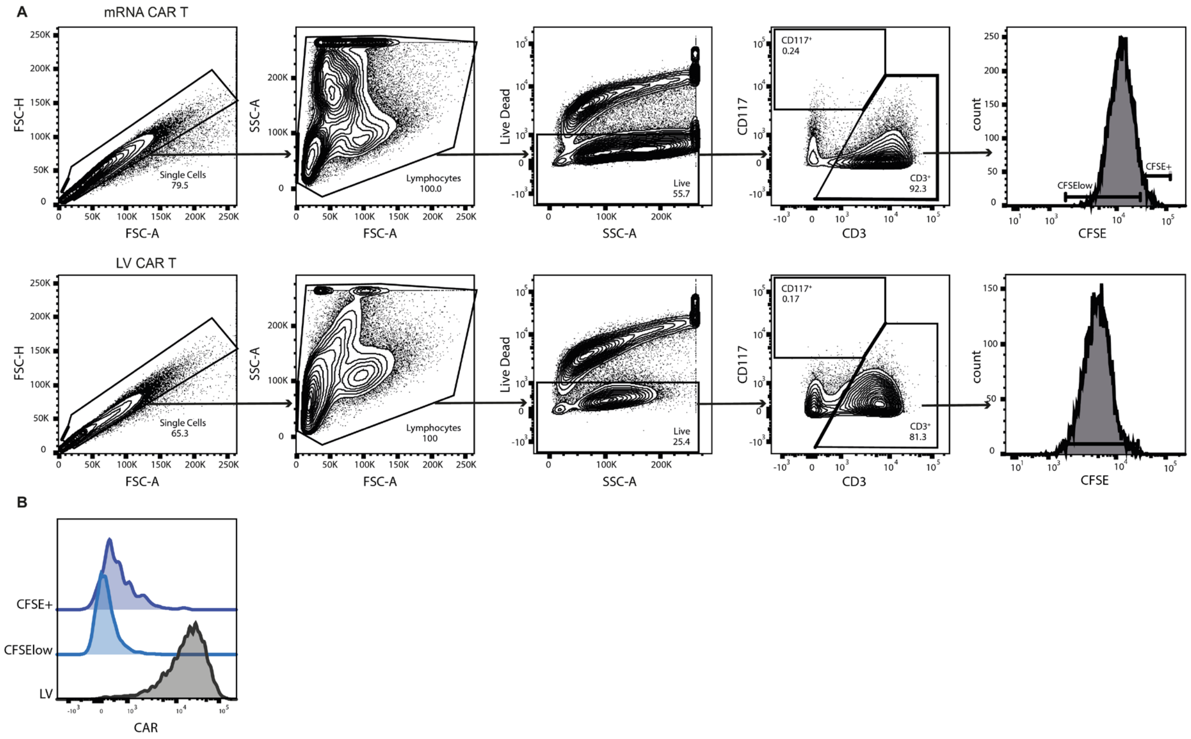

Figure S2: Ivt mRNA CD117 CAR T cells demonstrate efficient killing in vitro.
(A) CFSE stained T cells were co-culture with CD117+ HL-60 target cells for three days. Representative flow cytometric immunophenotyping showing the gating strategy in one representative donor (n = 3). Analyses were performed by gating into alive cells before evaluating lineage markers (CD117 and CD3) to retrieve target and effector cell percentages. (B) CAR expression of T cells gated according to CFSE retention in mRNA CAR T cells compared to LV CAR T cells. One representative histogram of results from three donors is shown.

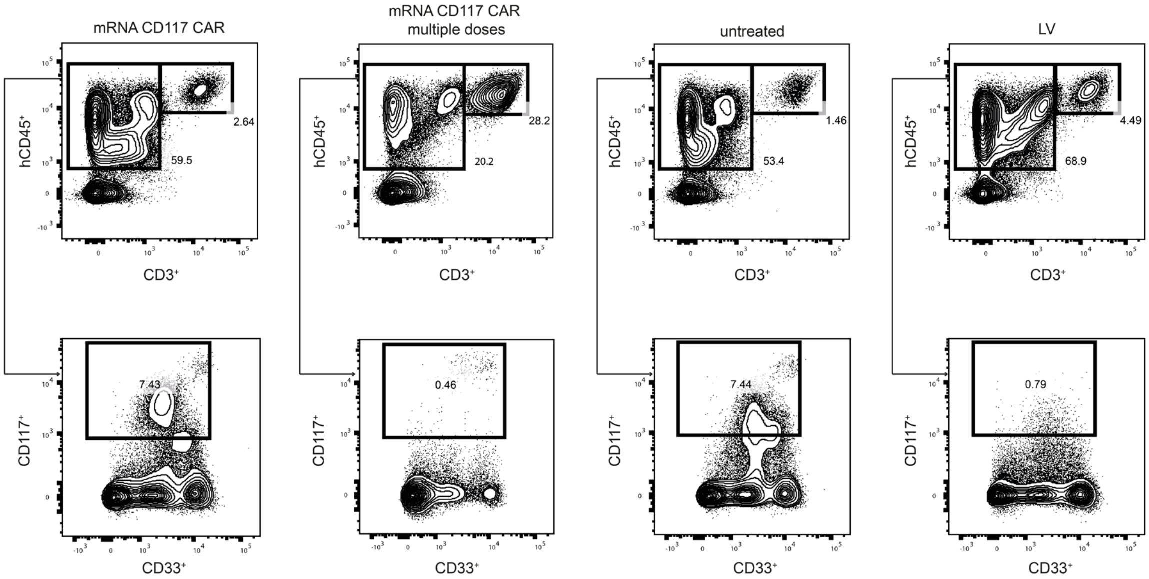

Figure S3: mRNA CD117 CAR T cells deplete healthy CD117 HSPCs in vivo.
Newborn NSG mice were sublethally irradiated and injected with CB-derived hCD34+ cells. After having confirmed the engraftment and establishment of human hematopoiesis, mice received a single dose of mRNA CAR T cells (2 x 10^6), two high doses mRNA CAR T cells (6 x 10^6 every three days), or a single dose of LV CAR T cells (2 x 10^6). Representative Flow cytometric immunophenotyping of the bone marrow of treated animals at endpoint. One representative mouse is shown (n=3).

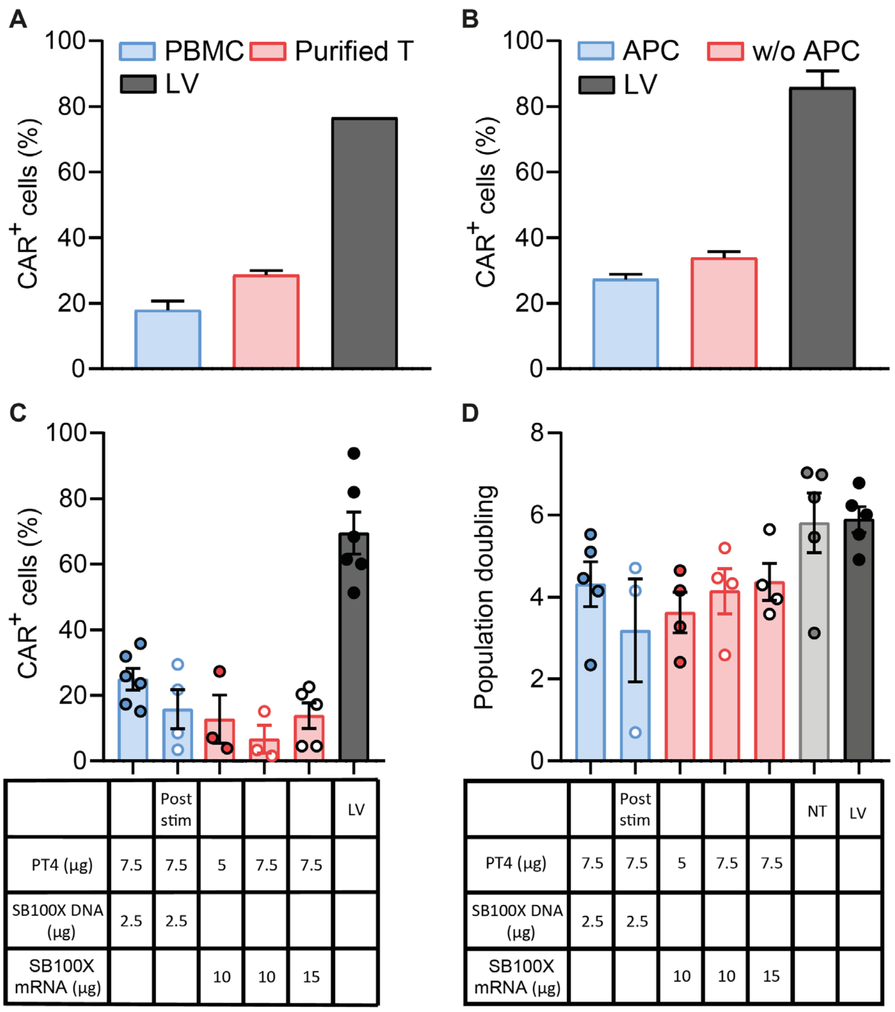

Figure S4: Transduction optimization of CD117 CAR T cells by SB vector.

(A) CAR T cells were generated by electroporation of total PBMCs or purified T cells in the presence of SB vector and the SB100X, compared to LV transduced purified T cells. CAR expression as percentage of CD3 cells as determined by flow cytometry with the recombinant c-Kit protein at 10 days after electroporation. (B) CAR expression as percentage of CD3 cells in T cells electroporated with SB vectors and stimulated in the presence or absence of APC, compared to LV transduced T cells. (C) CAR expression as percentage of CD3 cells of T cells electroporated with different concentrations of the PT4 vector and SB100X transposase, provided as DNA plasmid or mRNA, before or post stimulation, compared to LV transduced T cells, analyzed by flow cytometry at 10 days after electroporation. (D) Population doubling of T cells electroporated with different concentrations of the PT4 vector and SB100X transposase, provided as DNA plasmid or mRNA, before or post stimulation, compared to LV transduced T cells, analyzed by flow cytometry at 10 days after electroporation.

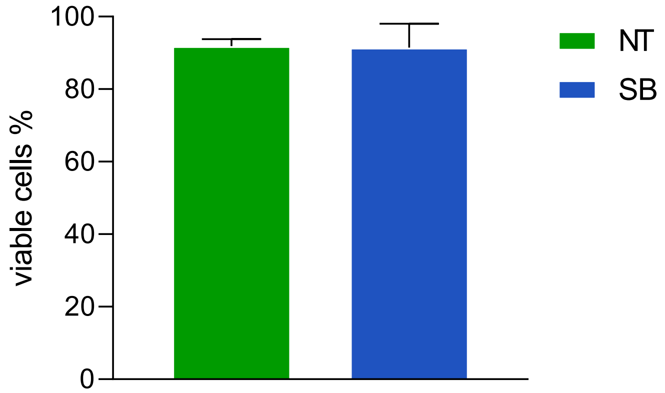

Figure S5: Viability of CD117 CAR T cells generated by the SB vector at 24 hours after electroporation. CAR T cells were generated by electroporation of purified T cells in the presence of the SB vector and the SB100X DNA and cell viability was assessed at 24 hours after electroporation. Data illustrate the mean ±SD from two different donors.

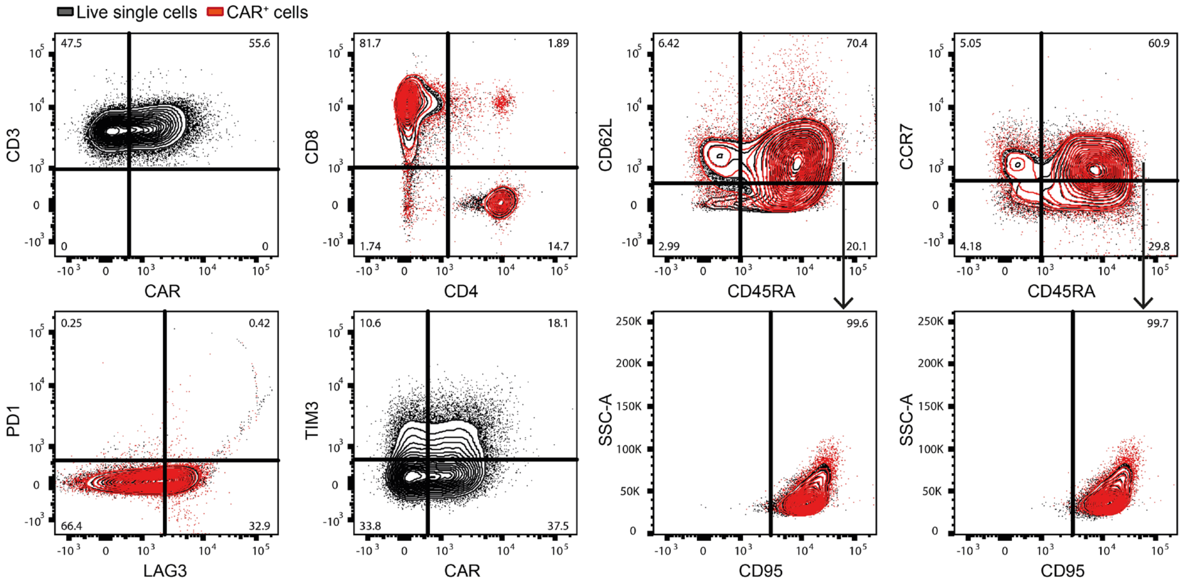

Figure S6: Phenotypical characterization of CD117 CAR T cells generated by SB vector.
CAR T cells were generated by electroporation of purified T cells in the presence of the SB vector and the SB100X. At the end of the 10-day differentiation protocol, cells were stained and acquired by flow cytometry. Dual-density plots in one representative batch are shown.

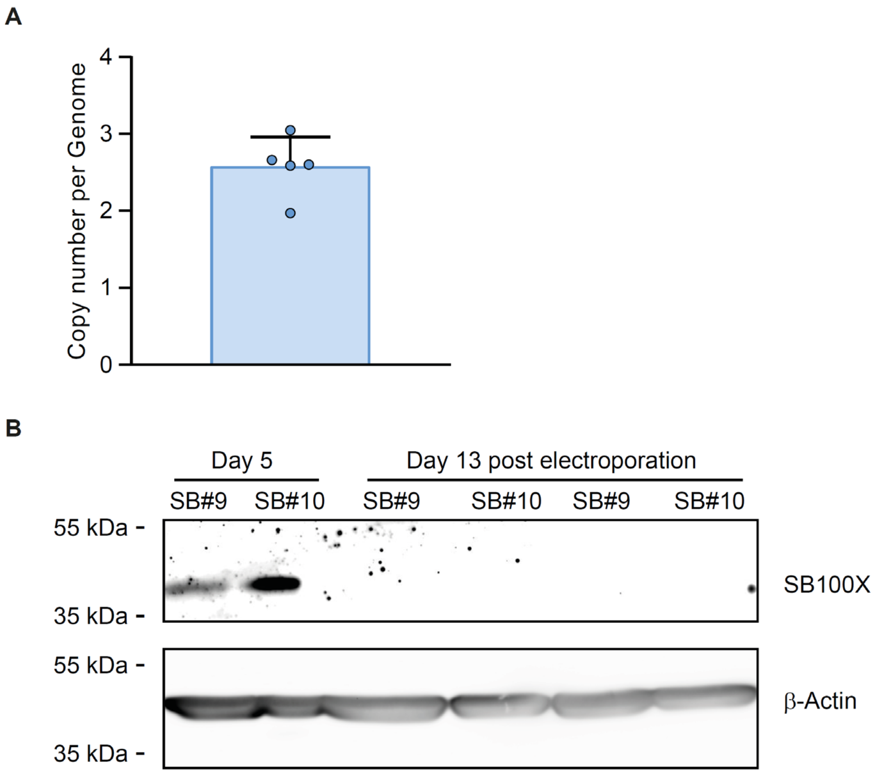

Figure S7: Transgene copy number and residual SB100X protein in CD117 CAR T cells generated by SB vector.

CAR T cells were generated by electroporation of total PBMC or purified T cells in the presence of SB vector and the SB100X. (A) Transgene copy number per genome as determined by digital PCR at 10 days after electroporation. (B) SB100X protein evaluated by western blot using protein extract isolated from NT and SB iC9.CAR T cells (SB100X DNA) at 5 and 13 days post electroporation. Data illustrate protein extract from 2 different donors, donor #9 and donor #10.

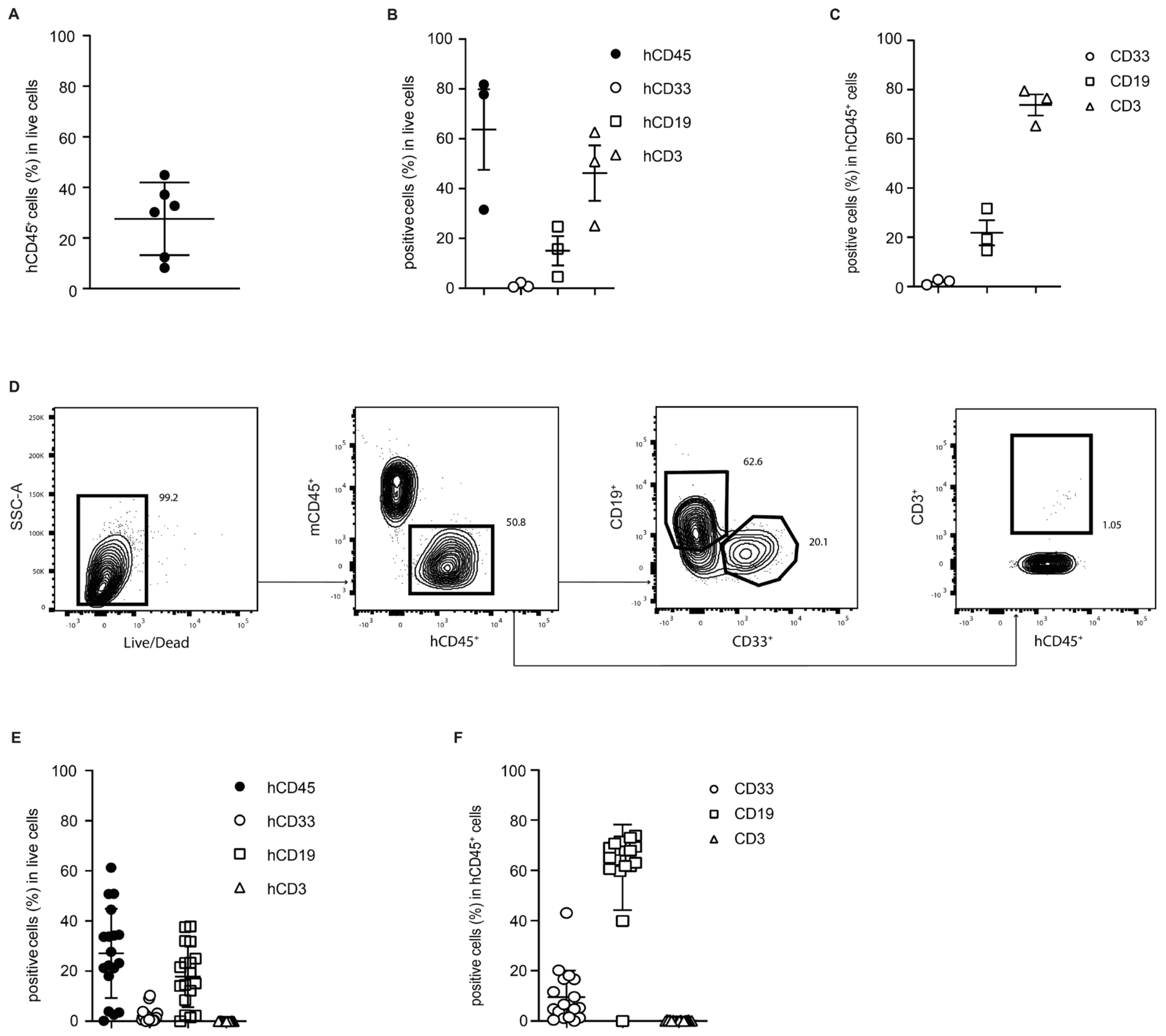

Figure S8: SB-engineered CD117 CAR T cells deplete healthy CD117 HSPCs in vivo.
(A) Human engraftment as percentage of hCD45 cells in live cells from peripheral blood of humanized mice, analyzed by flow cytometry at 40 days after transplantation. (B) Human engraftment as percentage of hCD45, hCD33, hCD19, and hCD3 cells in live cells from peripheral blood of humanized mice, analyzed by flow cytometry two weeks after CAR T-cell treatment (54 days after transplantation). (C) Human engraftment as percentage of hCD33, hCD19, and hCD3 cells in hCD45 cells from peripheral blood of humanized mice, analyzed by flow cytometry two weeks after CAR T cell treatment (54 days after transplantation). (D) Representative flow cytometric immunophenotyping with the gating strategy of the PB of mice engrafted with human CD34+ CB cells. One representative mouse is shown. (E) Human engraftment as percentage of hCD45, hCD33, hCD19, and hCD3 cells in live cells from peripheral blood of humanized mice, analyzed by flow cytometry at 60 days after transplantation. (F) Human engraftment as percentage of hCD33, hCD19, and hCD3 cells in hCD45 cells from peripheral blood of humanized mice, analyzed by flow cytometry at 60 days after transplantation.
