# Supplementary material for: Anti-CD117 CAR T cells incorporating a safety switch eradicate human acute myeloid leukemia and hematopoietic stem cells
Source: Mol Ther Oncolytics. 2023 Jul 19;30:56–71. doi: 10.1016/j.omto.2023.07.003 (PMC10424000; doi:10.1016/j.omto.2023.07.003)
Supplement: Document S2. Article plus supplemental information [file mmc2.pdf]

Molecular Therapy
Oncolytics
Original Article

Anti-CD117 CAR T cells incorporating a safety switch eradicate human acute myeloid leukemia and hematopoietic stem cells

Chiara F. Magnani,[1] Renier Myburgh,[1] Silvan Brunn,[1] Morgane Chambovey,[1] Marianna Ponzo,[2] Laura Volta,[1] Francesco Manfredi,[1] Christian Pellegrino,[1] Steve Pascolo,[3] Csaba Miskey,[4] Zoltán Ivics,[4] Judith A. Shizuru,[5] Dario Neri,[6] and Markus G. Manz[1]

[1]Department of Medical Oncology and Hematology, University Hospital Zurich and University of Zurich, Comprehensive Cancer Center Zurich (CCCZ), 8091 Zurich, Switzerland; [2]Tettamanti Center, Fondazione IRCCS San Gerardo Dei Tintori, 20900 Monza, Italy; [3]Department of Dermatology, University Hospital Zurich and University of Zurich, 8091 Zurich, Switzerland; [4]Division of Medical Biotechnology, Paul-Ehrlich-Institute, 63225 Langen, Germany; [5]Division of Blood and Marrow Transplantation, Department of Medicine, Stanford University, Stanford, CA 94305, USA; [6]Department of Chemistry and Applied Biosciences, Institute of Pharmaceutical Sciences, 8093 ETH Zurich, Switzerland

Discrimination between hematopoietic stem cells and leukemic stem cells remains a major challenge for acute myeloid leukemia immunotherapy. CAR T cells specific for the CD117 antigen can deplete malignant and healthy hematopoietic stem cells before consolidation with allogeneic hematopoietic stem cell transplantation in absence of cytotoxic conditioning. Here we exploit non-viral technology to achieve early termination of CAR T cell activity to prevent incoming graft rejection. Transient expression of an anti-CD117 CAR by mRNA conferred T cells the ability to eliminate CD117+ targets *in vitro* and *in vivo*. As an alternative approach, we used a Sleeping Beauty transposon vector for the generation of CAR T cells incorporating an inducible Caspase 9 safety switch. Stable CAR expression was associated with high proportion of T memory stem cells, low levels of exhaustion markers, and potent cellular cytotoxicity. Anti-CD117 CAR T cells mediated depletion of leukemic cells and healthy hematopoietic stem cells in NSG mice reconstituted with human leukemia or CD34+ cord blood cells, respectively, and could be terminated *in vivo*. The use of a non-viral technology to control CAR T cell pharmacokinetic properties is attractive for a first-in-human study in patients with acute myeloid leukemia prior to hematopoietic stem cell transplantation.

INTRODUCTION

Acute myeloid leukemia (AML) has a dismal prognosis due to the high rate of relapse, even after allogeneic hematopoietic stem cell transplantation (alloHSCT), which represents the therapy with highest curative potential for patients with adverse-risk disease.[1] AML arises from the accumulation of mutations in hematopoietic stem and progenitor cells (HSPCs), leading to the emergence of a population of malignant leukemia-initiating cells (LICs).[2–4] AML-LICs maintain high phenotypic similarity to their cells-of-origin and persisting LICs are sources of post-treatment disease relapse. Successfully targeting AML-LIC is therefore essential for AML cure. However, similar or same target expression on cell-of-origin HSPCs bares the risk and consequence of healthy HSPC depletion and subsequent severe myeloablation.[5]

Chimeric antigen receptor (CAR) T cell immunotherapy is a powerful treatment strategy that utilizes genetic engineering to enhance T cell antitumor activity through ectopic expression of a receptor recognizing tumor-associated antigens.[6,7] MHC-unrestricted recognition of antigens widely expressed on tumor cells and a multifactorial, self-sustaining T cell-mediated immune response are among the keys to the proven success in B cell malignancies, which has led to the approval and commercialization of several CAR T cell products in less than 5 years. In all of the approved instances, the surface targets are not specific for malignant cells but are also expressed on the cell-of-origin, i.e. B and plasma cells, which both are not critical for the immediate survival of patients and that might re-grow from their upstream progenitor cells. Using a similar approach targeting cell-of-origin antigens in AML-LIC will lead to long-lasting or permanent myeloablation. Indeed, most AML antigens currently targeted by CAR T cells, such as CD123,[8] CD33,[9] and CLL-1,[10] are expressed by HSPCs. Therefore, they are currently being evaluated mainly in a bridge-to-transplant setting, where hematopoiesis is subsequently rescued by alloHSCT.[11–13] This underlines the importance of either identifying specific AML-LIC targets or design strategies to mitigate expected toxicities.

We recently described the use of CAR T cells specific for the CD117 antigen to deplete LIC and replace HSPC by alloHSCT.[14] CD117, also

Received 13 January 2023; accepted 17 July 2023;
https://doi.org/10.1016/j.omto.2023.07.003.

Correspondence: Chiara F. Magnani, Department of Medical Oncology and Hematology, University Hospital Zurich and University of Zurich, Comprehensive Cancer Center Zurich (CCCZ), 8091 Zurich, Switzerland.
E-mail: chiara.magnani@usz.ch

 © 2023 The Authors.
This is an open access article under the CC BY license (http://creativecommons.org/licenses/by/4.0/).

referred to as c-Kit, is the receptor for stem cell factor (SCF), regulating HSPC self-renewal, quiescence, and survival in healthy and malignant hematopoiesis.[15–18] Depletion of CD117+ cells may enable concomitant AML-LIC and HSPC eradication, facilitating bone marrow niche clearance and subsequent alloHSCT in absence of genotoxic preconditioning.[19] This concept implies early termination of CAR T cell activity to prevent subsequent graft rejection.

In the current study, we therefore exploit several non-viral technologies for the generation of engineered T cells temporarily expressing anti-CD117 CAR molecules as well as safety switches to pre-clinically test their efficacy and termination. Our data support clinical testing of anti-CD117 CAR T cells in patients with relapsed AML and myelodysplastic syndrome (MDS).

RESULTS

Transient anti-CD117 CAR T cells generated by mRNA electroporation

mRNA incorporation into cells leads to transient protein expression. To improve the safety of anti-CD117 CAR T cells and allow for subsequent HSCT engraftment, we used mRNA electroporation to generate transient anti-CD117 CAR T cells. The protocol to deliver mRNA in T cells was established by using nucleofection technology, which we have previously used to generate transposon-based non-viral CAR T cells for B-ALL patients[20] and which allows highly efficient transfection (Figure 1A). Purified T cells were activated for 4 days and subsequently electroporated with CD117 CAR-encoding *in vitro* transcribed (ivt) mRNA modified with pseudouridine and 5-methylcytosin (m5C). We used a previously published anti-CD117 single-chain fragment variable (scFv)[14] generated from the sequence of the 79D antibody.[21] The CAR also included the hinge and transmembrane domains of the CD8 alpha chain, the 41BB costimulatory domain, and the CD3ζ domain. Electroporation of T cells with mRNA CAR minimally affected the CD4/CD8 ratio and the memory phenotype of CAR T cells compared with control T cells (Figure S1). The decay kinetics of CAR expression was evaluated over time by flow cytometry on total electroporated T cells, by labeling with *Staphylococcus aureus* protein A, which binds to antibody fragments featuring a variable heavy chain domain originating from the VH3 family[22] (Figures 1B and 1C) or, alternatively, with a target c-kit antigen (Figure 1D). High level of CAR expression was seen at 24 hours after ivt mRNA electroporation, with a mean fluorescence intensity (MFI) and a percentage of CAR-positive cells comparable to the one achieved by using a CD117 CAR-encoding lentiviral vectors (LV). A progressive decrease of CAR expression was seen over time and was more evident when 1.5 μg of mRNA CARs were electroporated compared with 10 μg, showing a persisting CAR expression, thought at low level, until day 3 after electroporation (Figure 1E). We therefore selected the electroporation with 10 μg of mRNA CAR for subsequent evaluation of CAR T cell functionality. T cell viability was 73.9% (±6.2%) and 87.6% (±0.8%) at 1 and 3 days post electroporation, respectively (Figure 1F). Functionality of T cells electroporated with mRNA CAR was assessed at day 1, day 2, and day 3 post electroporation. Cytotoxic assays on HL-60 cell lines transduced to express CD117 in comparison with wild-type (WT) HL-60 cell line confirmed specific killing of antigen-positive cell lines by T cells electroporated with ivt mRNA CAR and absence of activity toward the CD117 negative target. Cytotoxic activity decreased over time and was correlated with CAR expression kinetics (Figure 1G). Furthermore, to evaluate CAR expression in parallel to antigen-driven proliferation, T cells electroporated with mRNA CAR were stained with 5- (and 6-) Carboxyfluorescein diacetate succinimidyl ester (CFSE) and co-cultured with target cells for 72 hours. Progressive loss of CAR expression was associated with CSFE dilution and cell division, with complete absence of CAR expression on high-proliferating $CSFE^{low}$ cells. The absence of CAR expression was not associated with increased death of ivt mRNA CAR T cells compared with LV-transduced CAR T cells, indicating cell division as the primary mechanism of decreased ivt mRNA CAR expression. Complete elimination of the CD117+ target cells was observed at the end of the co-culture, indicating that transient CAR expression is sufficient for target cell eradication (Figures 1H, S2A, and S2B). Having demonstrated potent *in vitro* activity of transient anti-CD117 CAR T cells, we evaluated their *in vivo* activity against CD117+ healthy human HSPCs. Transplantation of cord blood (CB)-derived purified hCD34+ cells into irradiated newborn immunodeficient mice through intrahepatic injection allows for generation of humanized mice models with lymphohematopoietic reconstitution. After establishment of human hematopoiesis in NSG mice, we treated the mice with a single dose of 2×10^6 or two high doses of 6×10^6 ivt mRNA CAR T cells (Figure 1I). In contrast to treatment with a single dose of CAR T cells, two subsequent high doses (3 days apart) resulted in CD117+CD33+ cell depletion in the bone marrow (BM), similarly to mice treated with CAR T cells produced by LV vectors. In parallel with the target cell elimination, accumulation of CD3+ cells in the BM was observed (Figures 1J, 1K, and S3).

Collectively, these data show that electroporation of T cells with anti-CD117 CAR-encoding mRNA leads to transient CAR expression and concomitant acquisition of *in vitro* effector functions, which are progressively lost after T cell proliferation. In addition, transient expression of the CAR molecule induces specific activity *in vivo*, but requires infusion of at least two high doses of CAR T cells.

Production and characterization of non-viral anti-CD117 CAR T cells including the iC9 switch by SB vector

Although T cells transiently expressing anti-CD117 CAR from electroporated ivt mRNA have shown attractive features, such as efficacy and transient, self-terminating CAR expression upon proliferation, the need of at least two high doses may compromise feasibility for translation to the clinic. In order to facilitate the development of an anti-CD117 CAR strategy, we thus designed a genomically integrating vector based on a pT4 Sleeping Beauty (SB) transposon,[23] that includes the inducible Caspase 9 (iC9) switch and the anti-CD117 CAR, separated by a 2A peptide,[24] under the control of the human elongation factor 1 alpha (hEF-1α) promoter. SB allows the generation of CAR T cells for clinical application with demonstrated potent anti-leukemic activity.[20] iC9 allows rapid termination of CAR T cells

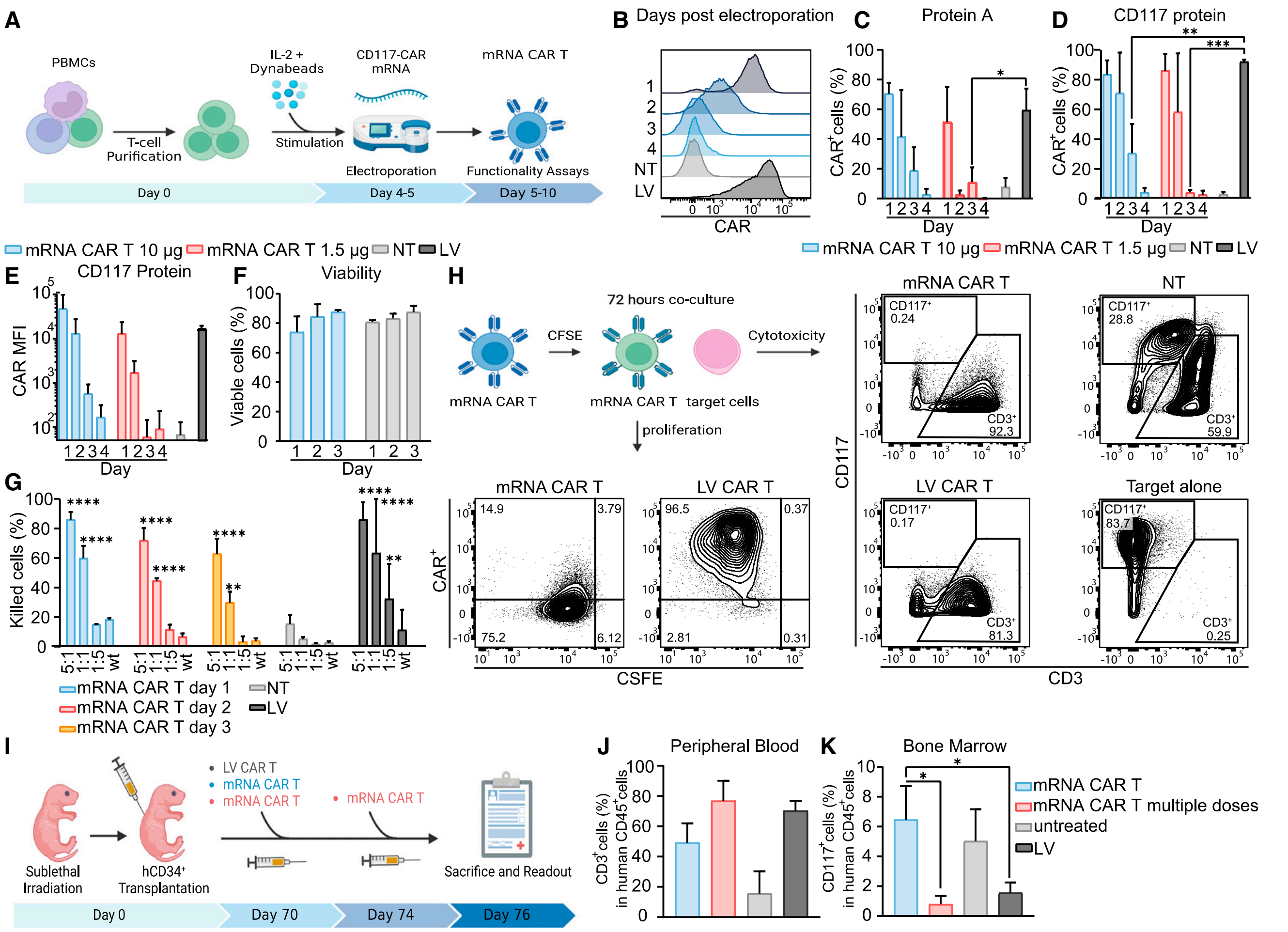

Figure 1. Transiently expressed anti-CD117 CAR T cells demonstrate efficient killing *in vitro* and *in vivo*

(A) Schematic representation of the method to generate mRNA CAR T cells. (B) CAR expression of T cells electroporated with 10 µg of CAR mRNA over time. One representative histogram of results from five donors is shown. (C) Percentage of CAR+ cells at different time points after electroporation as determined by flow cytometry with protein A. Two-way ANOVA with Turkey multiple comparison. (D) Percentage of CD3+CAR+ cells at different time points after electroporation as determined by flow cytometry with the recombinant c-Kit protein. Two-way ANOVA with Turkey multiple comparison. (E) CAR expression level (geometric mean) at different time points after electroporation as determined by flow cytometry with the recombinant c-Kit protein. Data illustrate the mean ± SD from five different donors for 10 µg, LV and NT cells, and three donors for 1.5 µg. (F) Viability of T cells electroporated with 10 µg of CAR mRNA over time as determined by Annexin V and 7AAD staining. Data illustrate the mean ± SD from three different donors. (G) Cytotoxicity of mRNA CAR T cells at different time points after electroporation compared with NT and LV CAR T cells against CD117+ HLA-60 at different E:T ratios and WT HL-60 (E:T = 1:1). Data illustrate the mean ± SD from three different donors. Two-way ANOVA with Turkey multiple comparison (*compared with NT cells). (H) Schematic outline of the co-culture experiment with CFSE-stained T cells. Representative flow cytometric immunophenotyping showing elimination of target cells (cytotoxicity, gated in live cells), and proliferation of CAR T cells (gated in CD3+ cells). One representative donor of results from three donors is shown. (I) Schematic outline of the *in vivo* experiment. (J) Percentage of CD3+ cells in PB of all treated groups at endpoint. (K) Percentage of BM CD33+CD117+ cells of all treated groups at endpoint. Data illustrate the mean ± SD from three different mice for mRNA CAR T cells. One-way ANOVA with Turkey multiple comparison.

by activation of the apoptotic pathway through the application of a small molecule that acts as a chemical inducer of dimerization (CID).[25] The resulting vector has an optimized donor vector architecture, has a full size of 8 kb, consists of an integrated cassette of 5 kb, and allows for the stoichiometric expression of the two transgenes (Figure 2A). Our previous LV vector including the CD117 CAR and RQR8[26] was used in parallel to compare SB-generated CAR T cells with CAR T cells transduced with LV. The hyperactive SB100X transposase, supplied as plasmid DNA or mRNA, catalyzes transgene integration.[27,28]

With the purpose of transfection optimization, we compared total peripheral blood mononuclear cells (PBMCs) and negatively selected bead-purified T cells as starting material. Total PBMCs were stimulated with anti-CD3 antibody OKT3 as we did for producing CAR T cells for clinical application (NCT03389035). Purified T cells

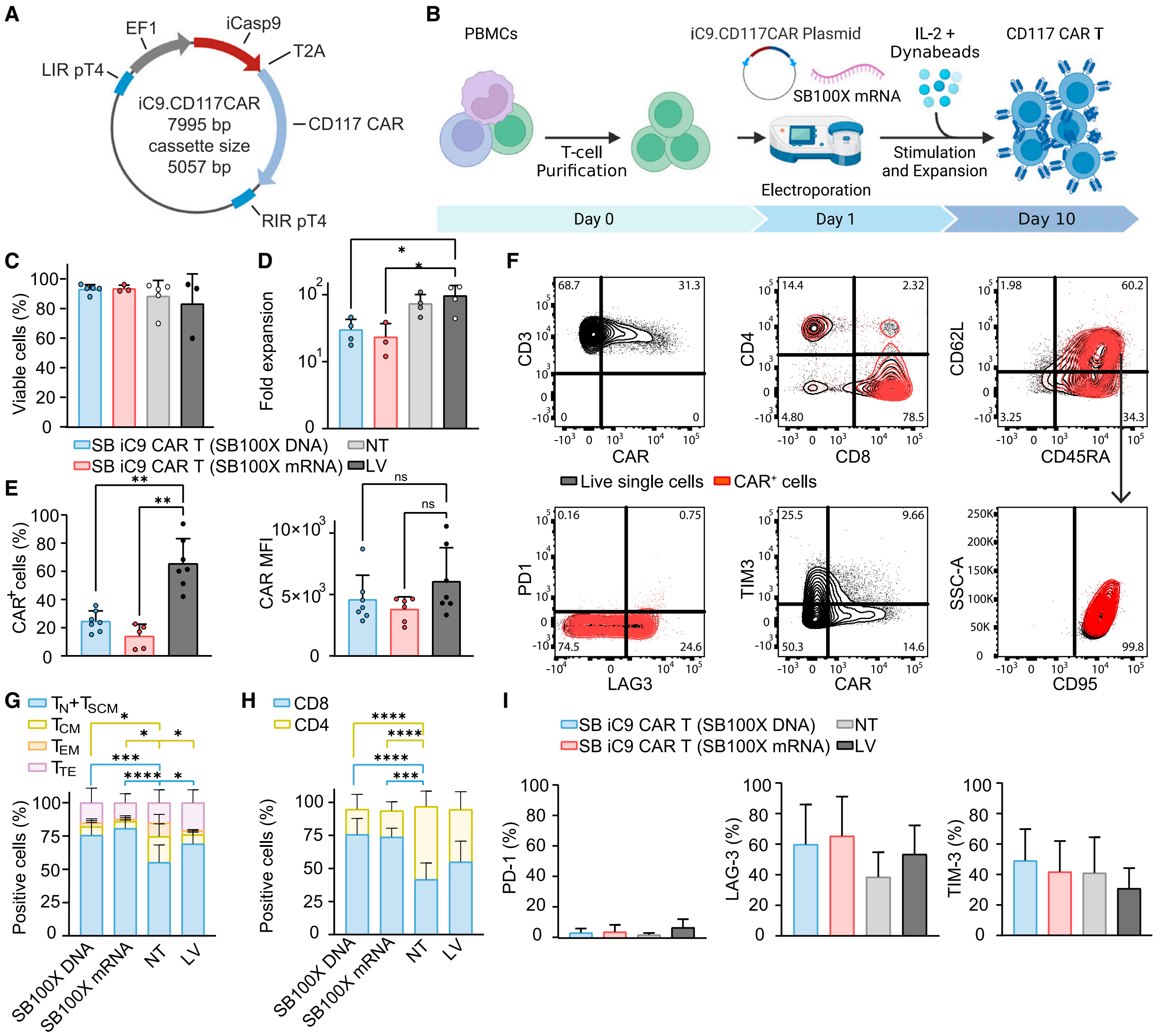

Figure 2. Generation and characterization of anti-CD117 CAR T cells incorporating iC9 by SB vector

(A) Schematic representation of the SB vector containing a bicistronic construct with the iC9 and anti-CD117 CAR transgenes under the regulation of hEF-1α. The transgenes are linked by a T2A peptide. The expression cassette is enclosed in the PT4 left/right inverted repeats/directed repeats (LIR/RIR). (B) Schematic representation of the method to generate SB CAR T cells. CAR T cells were produced by electroporation with the SB vector and the SB100X transposase. (C) Cell viability at the end of the manufacturing process. (D) Fold expansion calculated by dividing the number of total viable cells at the end of the expansion phase by the respective number on day 0. (E) CAR expression as percentage of CD3 cells and expression level (geometric mean) as determined by flow cytometry with the recombinant c-Kit protein. (F) Flow cytometric immunophenotyping by dual-density plots in one representative batch (n = 6). (G) Percentage of CD3+CD45RA+CD62L+CD95+ naïve-like (T_N) and stem cell memory (T_{SCM}), CD3+ CD45RA−CD62L+ central memory (T_{CM}), CD3+CD45RA−CD62L− effector memory (T_{EM}), and CD3+CD45RA+CD62L− terminal effector (T_{TE}). (H) Percentage of CD3+CD8+ and CD3+CD4+. (I) Percentage of CD3+PD-1+, CD3+TIM-3+ and CD3+LAG-3+. Data illustrate the mean ± SD from six different donors. Two-way ANOVA with Turkey multiple comparison (*compared with NT cells).

were stimulated with anti-CD3/CD28 paramagnetic beads and standard LV transduction was performed in parallel.[14] The protocol achieved T cell transfection with electroporation of total PBMCs as well as purified T cells (Figure S4A). Since in the clinical protocol we stimulated T cells with an autologous irradiated PBMC feeder after electroporation as a source of antigen-presenting cells (APC), we evaluated whether the addition of APCs can be avoided by stimulating purified T cells the day after electroporation with beads. T cell transfection was obtained in both conditions, in the presence or in the absence of PBMCs as a source of APCs (Figure S4B).

Furthermore, we performed electroporation in the presence of different concentrations of CAR transposon and SB100X plasmids or by using SB100X coding ivt mRNA. We also tested a condition with electroporation performed 2 days after stimulation. In our hands, electroporation of purified T cells with 7.5 μg of CAR transposon vector and 2.5 μg of SB100X DNA, followed by bead stimulation, allowed for the highest transduction efficiency (Figure S4C) and a favored *in vitro* expansion calculated as population doubling (Figure S4D) at the end of a 10-day *in vitro* culture (Figure 2B). SB100X could also be provided with ivt mRNA, particularly using the highest tested dose of 15 μg for 5×10^6 total T cells.

SB iC9.CAR T cells generated with the optimized conditions had a high level of viability (mean 93.1% ± SD 3.0%; range, 88.0%–96.4% for CAR T cells generated with SB100X DNA, and mean 93.5% ± SD 2.3%; range, 92%–96.2% for CAR T cells generated with SB100X mRNA) (Figures S5 and 2C), and a mean fold T cell number increase of 29.9 (range, 17.6–46.0) after 10-day expansion, supporting the production of clinically relevant numbers of non-viral CAR T cells (Figure 2D). CAR T cells showed stable CAR expression that was characterized by a MFI similar to the one achieved after LV-mediated transfection (Figure 2E). To understand whether gene transfer methods affect the final T cell composition, we performed extensive phenotyping at the end of differentiation. Notably, the procedure of generating CAR T cells with SB transposon did not affect the memory differentiation of T cells (Figure 2F). SB iC9.CAR T cells retained a higher proportion of CD45RA + CD62L + naïve-like population with features of T stem cell memory (mean 75.6% ± SD 9.6%, and mean 80.7% ± SD 6.4% for CAR T cells generated with SB100X DNA and SB100X mRNA, respectively), counterbalanced by a lower percentage of CD45RA−CD62L+ central memory (mean 6.4% ± SD 5.6%, and mean 5.3% ± SD 4.2% for CAR T generated with SB100X DNA and SB100X mRNA, respectively) in comparison with non-transduced (NT) cells (Figures 2G and S6). There was an increase in CD8/CD4 proportion in SB-generated CAR T cells compared with CAR T cells transduced with LV (mean of CD8+ cells 75.6% ± SD 12.0% and 73.8% ± SD 6.7% for CAR T cells generated with SB100X DNA and mRNA, respectively, compared with 64.9% ± SD 15.7% in LV-transduced CAR T cells (Figure 2H). Moreover, CAR T cells generated with SB transposon showed low levels of the exhaustion markers PD-1 (mean 3.0%, SD 2.8%), LAG3 (mean 59.9%, SD 26.1%), and TIM3 (mean 49.2%, SD 20.6, Figure 2I). In sum, these results demonstrate the potential of non-viral technology to efficiently deliver large multi-cistronic vectors and allow for simultaneous expression of multiple transgenes without affecting the viability and quality of primary T cells, supporting large-scale production and utilization at clinical level.

SB iC9.CAR T cells exhibit antigen-specific effector functions and iC9-mediated elimination of gene-modified cells

We subsequently tested whether generation of CAR T cells by non-viral engineering of a large, bicistronic cassette impacts on the functional activity *in vitro*. To evaluate their cytotoxic activity, SB iC9.CAR T cells were co-cultured for 24 hours with the AML cell line MOLM-14, which was transduced, and sorted to express human CD117, luciferase, and GFP. SB iC9.CAR T cells exhibited potent cytotoxic activity against the target, similar to cells engineered with LV encoding for the same CAR. Potent cytotoxicity was observed with unpurified CAR T cells at an Effector:Target (E:T) ratio of 1:1 (Figure 3A). Because the inferior amount of CAR+ T cells generated with SB compared with cells transduced with LV may lead to decreased functionality, we analyzed the level of pro-inflammatory cytokine production after target recognition in mixed CAR+ and CAR− T cells not purified for CAR expression. CAR T cells generated with SB or LV vectors did not differ in their ability to secrete interferon (IFN)-γ, tumor necrosis factor (TNF)-α, and interleukin (IL)-2 (Figure 3B). Potent cytotoxicity was observed with T cells sorted for CAR expression even in conditions where CAR T cells are at a disadvantage with respect to the target cells at E:T ratio of 1:5 (Figure 3C). Having proved specific and comparable effector activity of CAR T cells, we then evaluated if they are rapidly terminated by activation of the inducible Caspase 9. The addition of 200 nM of the CID AP20187 to cultures of anti-CD117 CAR T cells induced apoptosis of >95% of transduced CAR T cells within 24 hours, but had no effect on the viability of NT or LV cells (Figure 3D). This was also true with CAR+ sorted cells and even at low amount of the CID (Figure 3E). To evaluate whether target antigen expression affects CAR T cell activity, we used MOLM-14 cell lines modified to express different levels of CD117. Target antigen expression levels affected the kinetics of anti-CD117 CAR T cell killing, which was faster against MOLM-14 cell lines expressing high levels of the target antigen compared with target cells with medium and low levels (Figure 3F). No difference in cytotoxic activity was observed between SB IC9.CAR T cells and LV CAR T cells toward Molm-14 with high, medium, and low CD117 expression, indicating similar activation kinetics in cells produced with different transduction methods. Analysis of the transgene copy number of CAR T cells generated by SB revealed a mean of 2.5 (range 1.9–3) transgene integrations per cell (Figure S7A). Clearance of residual SB100X protein was confirmed in CAR T cells generated with SB100X DNA at the end of differentiation, assuring for the genomic stability of cellular product (Figure S7B). We conclude that SB iC9.CAR T cells are cytotoxic against the MOLM-14 cell line and are able to release cytokines. Delivery of an iC9 transgene by non-viral technology is feasible and functional to equip CAR T cells with a suicide gene as a safety measure.

SB iC9.CAR T cells show potent anti-leukemic activity *in vivo*

To evaluate the activity of iC9.CAR T cells in an AML mouse model, sub-lethally irradiated immunodeficient NOD.Cg-Prkdcscid Il2rgtm1Wjl/SzJ (NSG) mice were transplanted with 0.1×10^6 MOLM-14 cells modified to express high level of CD117 and luciferase. CAR T cells were injected intravenously at day 9 after MOLM-14 infusion, when all the mice had measurable sign of tumor engraftment (Figure 4A). CAR T cells are generally infused as bulk population for clinical application. Therefore, to evaluate the same dose of CAR T cells, we decided to infuse 2×10^6 CAR+ T cells per mouse, corresponding to 7.4×10^6 SB iC9.CAR T cells and 3.3×10^6 LV CAR T cells. As controls, mice were

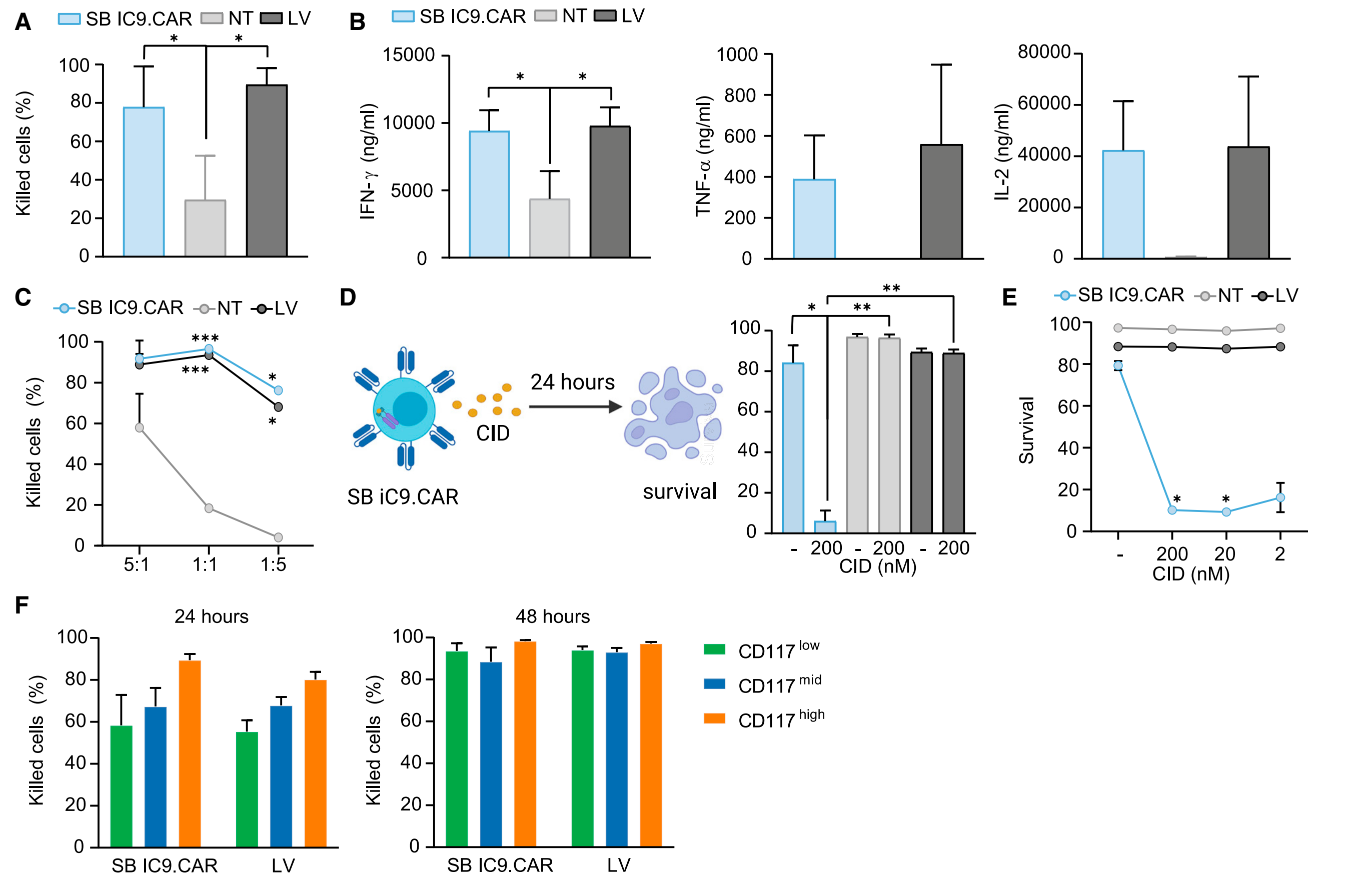

Figure 3. Anti-CD117.iC9 CAR T cells exhibit potent functionality and are rapidly terminated by iC9 activation

(A) Killing activity of CAR T cells after 24 hours of co-culture with CD117+ Molm-14 at an E:T ratio of 1:1. (B) IFN-γ, TNF-α, and IL-2 secretion of CAR T cells after 24 hours of co-culture with CD117+ Molm-14. (C) Killing activity of sorted CAR T cells at different E:T ratios. One representative experiment of results from three donors is shown. (D) Schematic outline of the co-culture experiment with CID. Apoptosis assessed in CAR T cells in the presence of 200 nM CID. (E) Apoptosis assessed in sorted CAR T cells at different CID concentrations. One representative experiment of results from three donors is shown. (F) Killing activity of sorted CAR T cells after 24 and 48 hours of co-culture with CD117+ Molm-14 expressing high, medium, and low levels of CD117 at an E:T ratio of 1:1. Data illustrate the mean ± SD from three different donors. One-way ANOVA with Turkey multiple comparison (*compared with NT cells).

infused with PBS or treated with 7.4 × 10^6 NT cells. SB iC9.CAR T cells mediated robust anti-leukemic activity *in vivo*, as demonstrated by the flux signal, leading to a significant survival advantage over control mice (median overall survival for NT = 22.5 days vs. SB = not reached, p = 0.0091, Mantel-Cox) (Figures 4B–4D). Notably, SB iC9 CAR T cells were as efficient as CAR T cells transduced with LV (median overall survival for NT = 22.5 days vs. LV = not reached, p = 0.0091, Mantel-Cox). At the time of euthanization, mice treated with SB iC9.CAR T cells showed almost complete clearance of leukemic blasts in the BM (Figure 4E) and spleen (Figure 4F). In this model, we observed a residual flux signal associated with circulating tumor blasts in the PB of three out of four mice treated with LV CAR and one out of four mice treated with SB iC9 CAR (Figure 4G). Taken together, these data indicate a comparably strong anti-leukemia activity in a xenograft model of human AML with LV and SB anti-CD117 CAR T cells.

Elimination of SB iC9.CAR T cells *in vivo* after HSPC depletion to enable subsequent alloHSCT

CD117 targeting is attracting increasing interest to eliminate healthy human HSPCs as a non-cytotoxic conditioning regimen before transplantation.[19,29] To examine the ability of SB iC9.CAR T cells to deplete healthy HSPC *in vivo*, irradiated newborn immunodeficient mice were reconstituted with human CD34+ CB cells (Figure 5A). On day 40 after transplantation, mice showed a mean of 27.6% hCD45+ PB cell engraftment, but absence of mature human cells in PB (Figure S8A). Mice then received 2 × 10^6 SB iC9 CAR T cells and were analyzed after 2 weeks of treatment. SB iC9.CAR T cells decreased CD117+ cell engraftment in the BM (1.1% ± 0.5%) compared with untreated animals, which showed a mean of 5.6% (±0.8%) of CD117+ HSPCs (Figure 5B). An increase of CD3+ cells in the BM of treated animals (Figure 5B) was associated with the observed reduction of CD117+ cells (Figures 5C and 5D). Notably, the presence of mature CD19+ B cells and CD33+ myeloid cells was

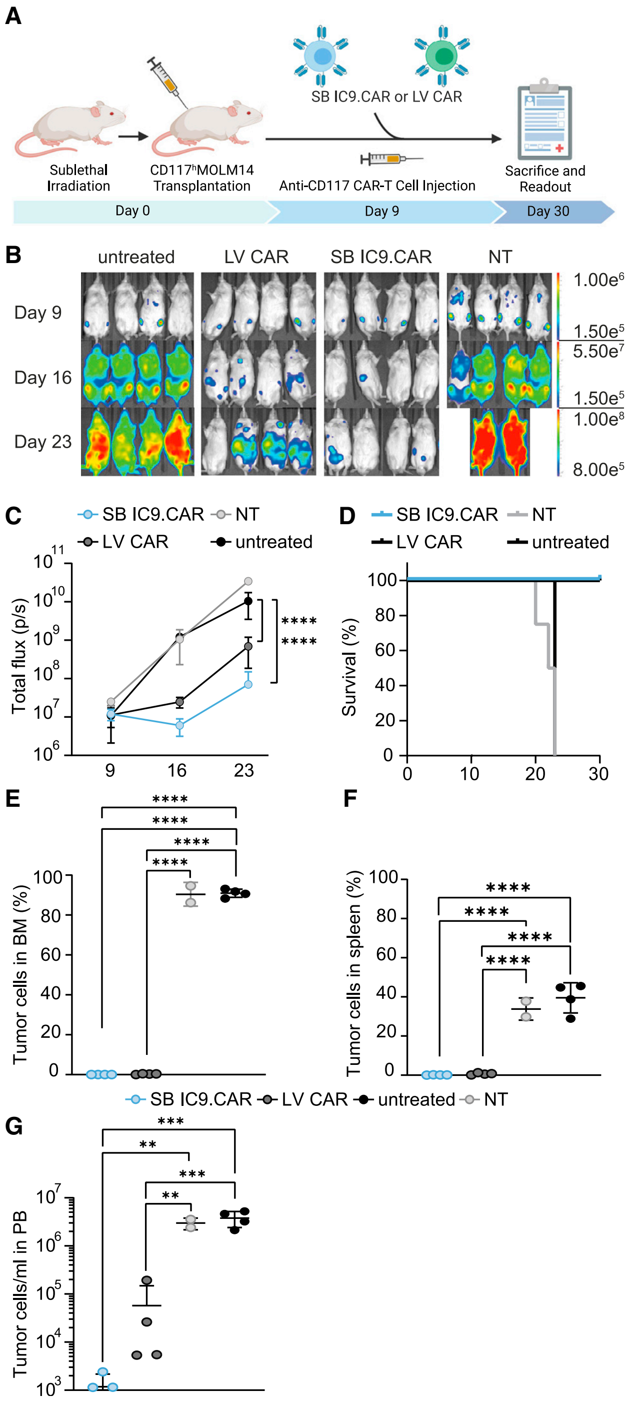

Figure 4. SB-generated anti-CD117 CAR T cells show potent anti-leukemic activity *in vivo*

(A) Schematic representation of the *in vivo* experiment. NSG mice were sub-lethally irradiated and injected with MOLM-14 CD117high Luciferase+ cells. At day 9, mice were injected with 2 × 10^6 CAR T cells, generated with SB or LV. (B) IVIS images of mice injected with CAR T cells, NT cells, or left untreated. (C) Bioluminescence intensity (total flux per second) over time in mice receiving the indicated treatment. Two-way ANOVA with Turkey multiple comparison (*compared with untreated). (D) Kaplan-Meier survival curves over time. Statistical differences are calculated by Mantel-Cox test. (E) Flow cytometry analyses of BM at euthanization, corresponding to day 20–23 for control groups (humane endpoints), and day 30 for groups treated with CAR T cells. (F) Flow cytometry analyses of spleen at euthanization. (G) Flow cytometry analyses of PB at euthanization. Data illustrate the mean ± SD from four different mice per group. One-way ANOVA with Turkey multiple comparison.

detected in the PB of mice treated with anti-CD117 CAR T cells 2 weeks after infusion, indicating the possibility to achieve complete elimination of CD117+ HSPCs before it results in myeloablation (Figures S8B and S8C).

Complete elimination of CD117+ HSPCs would allow clearance of malignant LICs and healthy HSPCs, but the persistence of CD117-targeting CAR T cells would compromise subsequent alloHSCT. Having demonstrated that SB iC9.CAR T cells efficiently target healthy CD117+ cells in humanized mice, we assessed the possibility to terminate CAR T cells after the complete elimination of CD117+ HSPCs. We consequently generated humanized mice by engrafting human HSPCs and waited 60 days for high human cell engraftment (mean 27.19% ± SD 17.8%) and reconstitution of mature human hematopoiesis (Figures S8D–S8F). In this setting, we evaluated CD117+ cell depletion after 7 and 14 days of treatment and compared once more the activity of non-viral CAR T cells with LV-engineered CAR T cells (Figure 5E). anti-CD117 CAR T cells were able to eliminate CD117+ HSPCs after 2 weeks of treatment (40.5% ± 12.64% and 0% ± 0% as mean engraftment in the control group compared to treated animals), while 1 week of treatment reduced the engraftment (2.46 ± 0.94) but was not sufficient to achieve complete CD117+ cell depletion (Figure 5F). High CD3+ engraftment was observed in the PB of treated animals (Figure 5G). To eliminate SB iC9.CAR T cells *in vivo*, mice were treated with a single dose of CID on day 17 after CAR T cell treatment. Since mice were infused with non-sorted CAR T cells, three doses of Antithymocyte Globulin (ATG) were provided on days 15, 16, and 17 to also eliminate the fraction of not transduced and thus non-iC9 expressing T cells, as it is usually included in alloHSCT conditioning regimen and was previously used by us to deplete CAR T cells *in vivo*.[14] Treatment with the combination of CID and ATG completely eliminated anti-CD117 CAR T cells and T cells (Figures 5H and 5I) in the PB of respective animals at day 5 after CAR T cell termination treatment. Overall, these results demonstrate the feasibility of completely eliminating anti-CD117 CAR T cells having mediated the healthy HSPC depletion, supporting their potential use as a conditioning regimen in alloHSCT. Moreover, we found that 2 weeks of treatment might be a favorable window of opportunity to achieve complete elimination of CD117+ HSPCs before it results in myeloablation and the perfect time for CAR T cell elimination and subsequent transplantation. The analysis did not show any significant difference in the CD117+ HSPC depletion between the groups treated with LV-engineered CAR T cells and CAR T cells generated with SB.

Activation of iC9 leads to CAR T cell functional clearance *in vivo*

To investigate whether activation of the iC9 safety switch would allow controlling CAR T cell function *in vivo*, we used tumor engraftment as

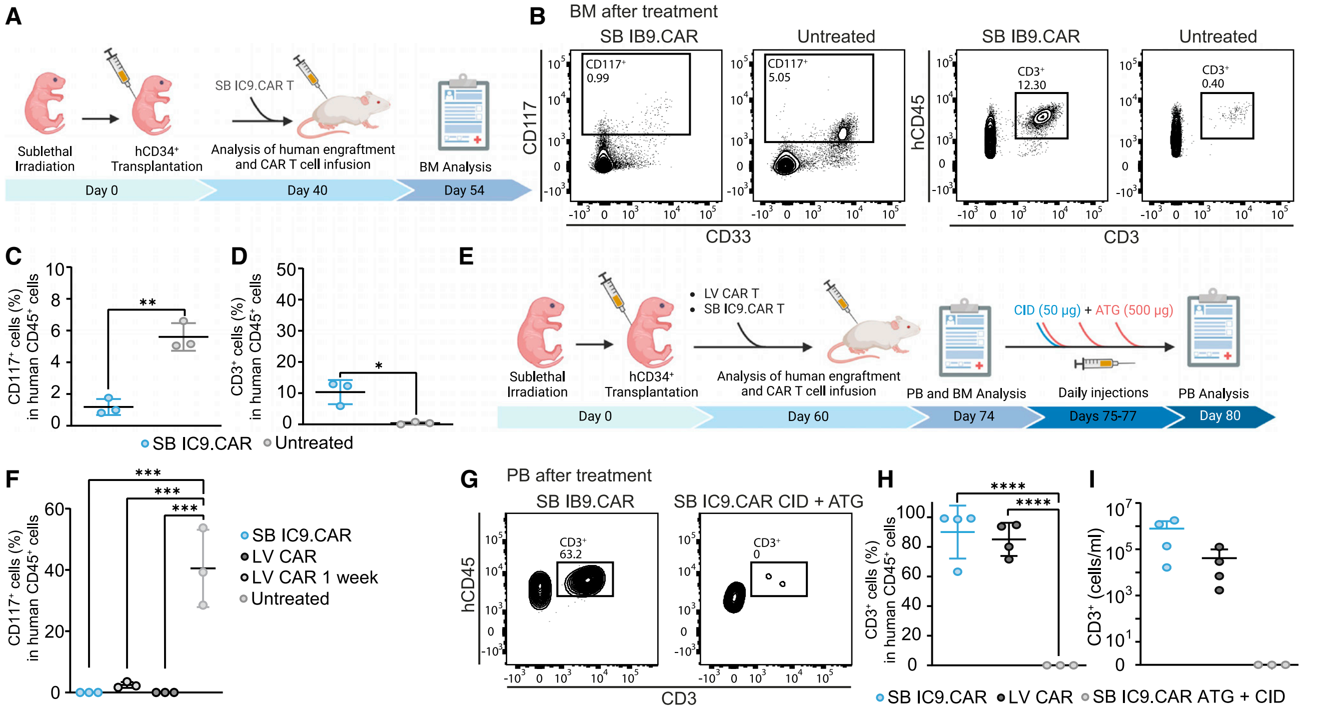

Figure 5. SB-generated CAR T cells are able to deplete human CD117+ HSPCs and are terminated by iC9 activation and ATG

(A) Newborn mice were sub-lethally irradiated and injected intrahepatically with human CB-derived CD34+ cells. Following engraftment at day 40, mice were injected with 2×10^6 CAR T cells generated with SB. (B) Representative flow cytometry plots of CD117+ and CD3+ cells in BM of animals from each treatment group after 2 weeks of treatment. (C) Percentage of CD117+ of hCD45+ cells in BM. (D) Percentage of CD3+ cells of hCD45+ cells in BM. (E) Newborn mice were sub-lethally irradiated and injected intrahepatically with human CB-derived CD34+ cells. Following engraftment at day 60, mice were injected with 2×10^6 CAR T cells generated with SB or LV. Two weeks after treatment, PB and BM analysis was performed by bleeding and BM puncture. Mice were then treated with a single dose of CID (50 µg/mouse) and three subsequent doses of ATG (500 µg/mouse) from day 75 to day 77. (F) Percentage of CD117+ cells of hCD45+ cells in BM after 1 or 2 weeks of treatment. (G) Representative flow cytometry plots of hCD3+ cells in PB of animals from each treatment group before and after CAR T termination. (H) Percentage of CD3+ cells of hCD45+ cells and (I) CD3+ cells/mL in PB before and after CAR T termination.

a surrogate marker for the functional absence of CAR T cells. Sublethally irradiated immunodeficient NSG mice were transplanted with MOLM-14 cells. Once the mice showed tumor engraftment, they were infused with 1.4×10^6 purified CAR+ T cells intravenously and, 3 days post-infusion, received CID or were left untreated. The activity of the iC9 safety switch in SB iC9.CAR T cells was evaluated in comparison to antibody-mediated elimination of LV CAR T cells by Rituximab/RQR8 switch (Figure 6A). As controls, mice were treated with NT cells. CID treatment abolished SB iC9.CAR T cell-mediated anti-leukemic activity *in vivo*, as demonstrated by the flux signal, indicating functional depletion of CAR T cells early after CID treatment (Figure 6B). CAR T cells became undetectable in PB 4 days after activation of the suicide switch in two out of four treated mice (Figure 6C). Their clearance was confirmed after 14 days in BM and spleen of treated animals and was associated with tumor engraftment. Rituximab-treated mice showed a significant reduction of CAR T cells in BM at the end of the experiment (Figure 6D). However, a longer time was apparently required to achieve depletion since CAR T cells were still visible 3 days after infusion in the NSG model (Figure 6C). Thus, LV CAR T cells were still functional and able to control the disease in Rituximab-treated mice (Figure 6D). Mice treated with CID were euthanized due to disease progression, and their survival was inferior to mice where CAR T cells have not been depleted and to Rituximab-treated mice as well (median overall survival for NT = 22 days, SB IC9.CAR = LV CAR = not reached, SB IC9.CAR + CID = 25 days and LV CAR + Rituximab = 26 days, SB IC9.CAR vs. SB IC9.CAR + CID p = 0.0027, Mantel-Cox, Figure 6E). In summary, the iC9 safety switch allowed for the rapid elimination of CAR T cells *in vivo* associated with loss of on-target efficacy, which can also be achieved by antibody-mediated depletion, although with a slower kinetic.

DISCUSSION

We aimed to develop a CAR T cell approach to target AML-LIC that could also be used as a conditioning regimen prior to alloHSCT. We showed that electroporation of an IVT mRNA encoding a CD117 CAR is feasible to generate functional T cells that temporarily express CD117 CAR molecules, exhibit transient cytotoxicity, and *in vivo* activity when infused in at least two high doses. Alternatively, stable expression of CD117 CAR and the iC9 suicide gene in T cells by using a genomically integrating SB vector led to CAR expression and *in vitro* CAR T cell activity toward CD117+ AML cells. Activation of the iC9 transgenes induced CAR T cell apoptosis, which would

allow for CAR T cell depletion before alloHSCT. Anti-CD117 CAR T cells engineered with the SB vector showed anti-leukemic activity in a human tumor xenograft model and completely depleted healthy HSPC in immunodeficient mice reconstituted with a human hematopoiesis. We observed no difference in activity when CAR T cells produced by a non-viral approach with the SB vector were compared with cells engineered with LV vectors. SB iC9.CAR T cells were terminated *in vivo* by activation of iC9.

AML-LICs derive from HSPCs with little immune-phenotypic distinction.[2,3,30] In contrast to mature lineage antigen-targeting in B-lineage and plasma cell neoplasia, there is no consensus on which surface antigen might be a suitable target to develop anti-AML therapies, eradicating LIC. We and others identified CD117 as potential target, as it is expressed by the CD34+CD38− and CD34+CD38+ cell fractions in healthy BM and CB, and in about 80% of AML blasts.[19] In contrast to myeloid markers as CD33, which are not required for cell survival,[31] CD117 might be of relevance for SCF-mediated LIC support,[32] and CD117 might thus be less prone to immune-pressure selected antigen loss. As antigens suitable for targeting the AML-LIC are commonly shared by healthy HSPCs and myeloid progenitors, myeloablation is inevitable and therefore we evaluated whether CAR T cells targeting CD117 would eliminate HSPCs *in vivo*. Flow cytometric analysis of CD117+ cells in BM of animals engrafted with human hematopoiesis revealed HSPC reduction after 1 week of infusion and complete eradication after 2 weeks. Likewise, the majority of anti-AML CAR T cell strategies have the concern to induce severe BM aplasia.[33] Preliminary clinical evaluation of anti-CD33 CAR T cells in patients with AML indeed showed on-target/off-tumor toxicity to HSPCs and myeloid progenitors, observed as pancytopenia after treatment.[34] Even though CLL-1 is expressed at lower levels by healthy HSPCs and has a higher specificity for AML-LIC, reduction of monocyte and neutrophil counts were observed in pediatric and adult patients treated with anti-CLL-1 CAR T cells, respectively, and may require bridging alloHSCT to rescue long-term on-target off-tumor toxicity.[35,36] Similar or even more severe BM aplasia is expected after CD117 targeting, thus making it necessary to replace hematopoiesis with alloHSCT after immunotherapy. To spare normal HSPCs and avoid alloHSCT, target antigens recognizing AML-LIC without HSPC toxicity such as CD70 have been proposed.[37] Nevertheless, CD70 antigen density on AML cells is below that needed to trigger CAR T cell response, which can only be achieved by increasing CAR binding avidity and pharmacological intervention.[38]

The use of an anti-AML CAR strategy as a bridge to HSCT approach implies the need to mitigate toxicities on the incoming graft, i.e. graft rejection. We previously demonstrated significant *in vivo* depletion of anti-CD117 CAR T cells with Rituximab using a vector incorporating the selection marker RQR8,[14] which combines target epitopes from CD34 and CD20 antigens.[26] However, depletion of CAR T cells was incomplete. To completely eliminate CAR expression, we tested the use of ivt mRNA to manufacture transiently active anti-CD117 CAR T cells, obviating the need to administer agents for elimination. Of note, mRNA technology has taken a huge step forward in recent years due to the Covid-19 mRNA vaccines, increasing the availability of platforms to generate mRNA for clinical application. In this study, we took advantage of recent progress in mRNA design,[39–41] and used modified RNA (pseudouridine and 5-methyl cytosine) as well as CleanCap[42] to improve translational capacity and diminish immunogenicity.[43] The optimized mRNA design in combination with an improved protocol to generate mRNA CAR T cells by electroporating T cells after stimulation resulted in heterogeneous >85% CAR expression, with minimal loss of viability. Compared with other protocols that manipulate resting T cells to avoid early loss of CAR expression,[44] our manufacturing protocol was faster and achieved similar kinetics in the acquisition of CAR-redirected effectors. Reducing the manipulation of CAR T cells *ex vivo* is known to preserve a less differentiated memory phenotype and effector activity, and indeed, most mRNA CAR T cells showed a central or effector memory phenotype. Furthermore, mRNA CAR T cells showed potent effector activity and eliminated resident HSPCs in a humanized mice model engrafted with human CD34+ CB-derived HSCs. However, an important limitation emerging from our *in vivo* preclinical results is the need of manufacturing multiple doses, which can be challenging as observed in clinical trials with mRNA CAR T cells.[45] This limitation might be overcome through *in vivo* delivery of mRNA with lipid nanoparticles.[46] However, this strategy may not be ideal for addressing a rapidly progressive disease such as AML, as suggested by the lack of demonstrated efficacy of the phase I study with anti-CD123 mRNA CAR T cells (NCT02623582).[47]

Based on these results, we combined stable transduced CAR T cells with the iC9 transgene, which guarantees rapid elimination of CAR T cells to avoid subsequent graft rejection upon administration of the inert and approved drug Rimiducin. Moreover, selective modulation of CAR T cell expansion can be achieved by titrating the dose of the CID, which allowed mitigation of toxicities such as immune effector cell-associated neurotoxicity syndrome after anti-CD19 CAR T cell treatment without interference on CAR T cell efficacy.[25] Inclusion of a safety switch will also allow for reduced intensity lymphodepletion before proceeding to transplantation, which is ideal due to common comorbidities among AML patients. CD117 is also expressed by mature mast cells,[48] interstitial cells of Cajal, melanocytes, tubular epithelial cells of the kidney, and certain cells in the reproductive organs.[14] Consequently, the presence of a switch that can induce apoptosis within minutes of administration and with a clinical proof of efficacy[49] might be beneficial to ensure safety and successful translation of this approach to the clinic. We demonstrated that the inclusion of iC9 in our anti-CD117 CAR vector allows for efficient apoptosis induction in anti-CD117 CAR T cells upon CID administration, without affecting CAR T cell effector functions evaluated *in vitro* and *in vivo* in murine xenograft models. Functional elimination of on-target CAR T cell activity was observed in mice engrafted with CD117+ Molm-14 cell lines after CID administration. Interestingly, mice show comparable expression of CD117 to humans in organs and tissues.[14] The absence of reported on-target non-hematopoietic toxicity in a murine anti-CD117 CAR in syngeneic mouse model[50] gives confidence that future safety evaluation will be feasible in the context of early-phase clinical trials in humans.

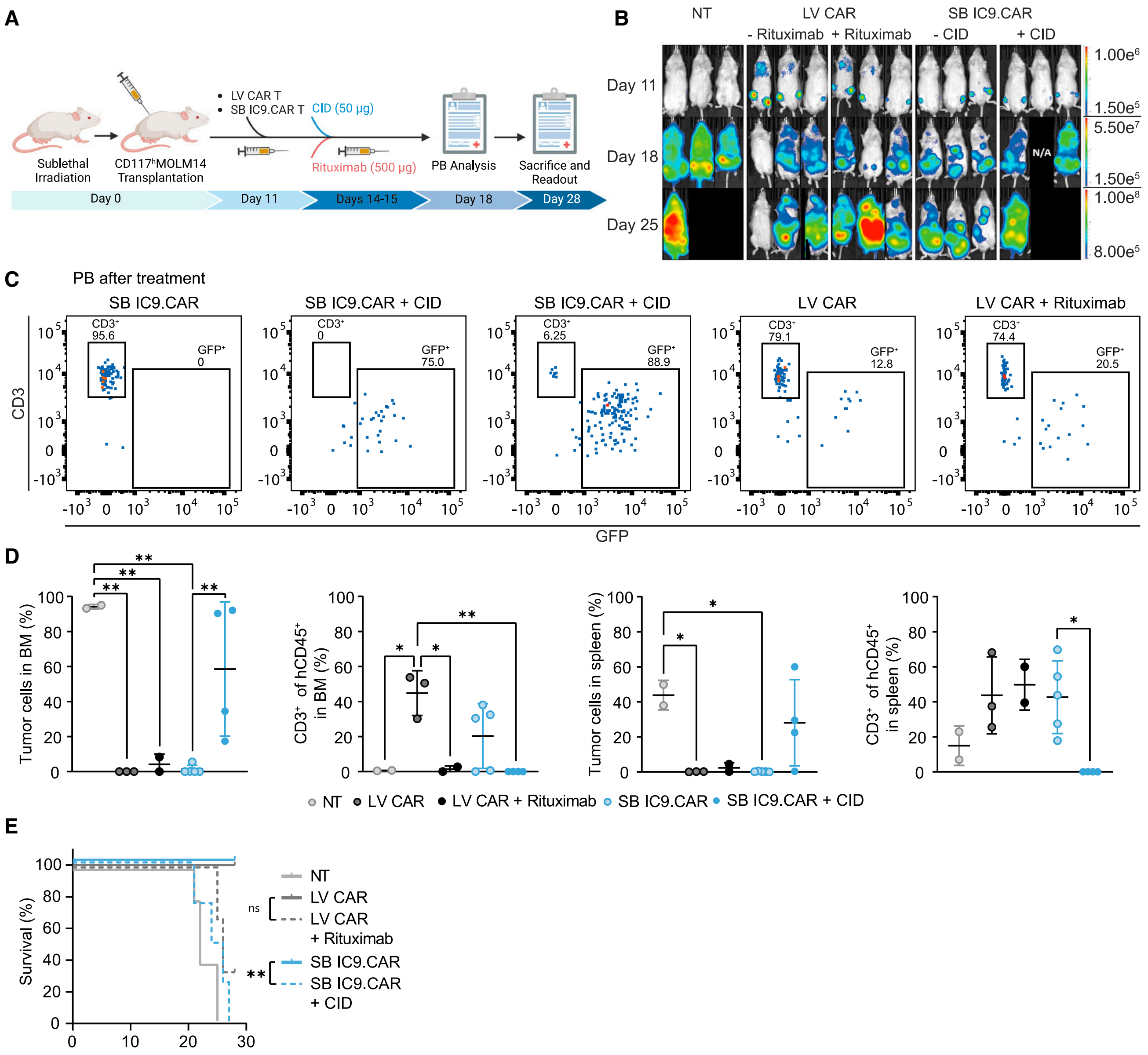

Figure 6. Functional demonstration of iC9-mediated CAR T cell clearance *in vivo*

(A) Schematic representation of the *in vivo* experiment. NSG mice were sub-lethally irradiated and injected with MOLM-14 CD117high Luciferase+ cells. Mice were injected with 1.4 × 10^6 purified CAR+ T cells, generated with SB encoding iC9 or LV encoding RQR8 safety switches at day 11 and, after 3 days, received the CID or Rituximab, respectively. (B) IVIS images of three representative mice injected with NT cells, LV RQR8.CAR T cells, or SB IC9.CAR T cells, and treated with Rituximab or CID. (C) Representative flow cytometry plots of hCD3+ cells and GFP+ tumor cells of animals from each treatment group assessed in PB on day 18. (D) Flow cytometry analyses of BM and spleen at euthanization. (E) Kaplan-Meier survival curves over time. Statistical differences are calculated by Mantel-Cox test. Data illustrate the mean ± SD from three different mice per group in NT, LV RQR8.CAR T cells, Rituximab; five different mice per group in SB IC9.CAR T cells; and four different mice per group in SB IC9.CAR T cells + CID. One-way ANOVA with Turkey multiple comparison.

An important observation that emerges from the data comparison is the feasibility of SB vector to allow for T cell engineering with complex and large gene cassettes compared with viral vectors that are limited in the genetic cargo capacity due to packaging size constraints. No significant differences in antitumor activity were identified between LV-transduced and SB-engineered anti-CD117 CAR T cells *in vitro* and *in vivo*. We only observed a peculiar increase in CD8/CD4 proportion in SB iC9.CAR T cells, despite using the same CAR design in terms of stimulatory domains and cytokines to expand CAR T cells, which may be due to SB itself or the electroporation procedure. As previously shown for CAR T cells engineered with SB and *piggyBac* transposons,[51] our SB iC9 CAR

T cells maintain a good proportion of T stem cell memory phenotype, which is associated with superior antitumor activity in mouse models.[52] Moreover, SB iC9 CAR T cells showed low levels of inhibitory receptors, which are known to contribute to T cell exhaustion and have been recently identified as predictors of cellular products associated with decreased response.[53] We previously demonstrated the proof of concept for the clinical safety and anti-leukemic efficacy of SB-engineered CAR T cells.[20] Now, taken together, these results suggest that our SB multi-cistronic vector can generate CAR T cell products with a T cell composition associated with T cell fitness and potent antitumor activity in xenograft models. Since the CAR T cell landscape is rapidly evolving toward an increased complexity in transgene design to allow for multi-targeting, our observation clearly positions SB as a valuable non-viral vector for future applications, also allowing for increased sustainability and access compared with LV transduction.[39]

An increasing number of preclinical studies in immunocompromised mice demonstrated the utility of CD117 depletion as immunologic conditioning, allowing for HSC niche clearance and engraftment of donor-derived HSCs.[54] CD117 antibody-mediated immunological conditioning has the advantage of avoiding the toxicity of cytotoxic regimens applied in alloHSCT, is currently being clinically evaluated in benign hematologic diseases, and has shown encouraging preliminary data in terms of safety and activity.[55] However, complete eradication of host HSCs is likely not achieved with anti-human CD117 antibodies,[29] indicating that increased potency may be needed for hematological malignancies to allow for eradication of host HSC/LIC. We proved that SB iC9 CAR T cells are able to completely eradicate healthy HSCs in mice reconstituted with a human hematopoiesis, allowing for niche clearance and thus possibly obviating the use of cytotoxic conditioning before transplantation in AML or MDS. Notably, our data indicate that CAR T cells have to persist 2 weeks for achieving complete HSC/LIC depletion, which may also lead to durable leukemia remission. As the mature circulating myeloid cells do not express CD117, ablation of HSC did not immediately result in myeloablation, indicating a possible time window for intervention in which the CAR T cells can remain active without lethal toxicity to the host. Unfortunately, our humanized model precludes the study of subsequent donor HSC engraftment because of competition with mouse HSPCs and possible rejection/GVHD by mature human T cells from the previous graft.[56] For the clinical application, we envision co-administration of CID and reduced intensity lymphodepleting regimen, such as ATG, which is commonly used in the clinic before transplantation, but might interfere with the SCID-repopulating ability of human CD34 cells in mouse xenografts.[57] Using CID and ATG, we proved effective SB iC9.CAR T cell termination and complete T cell depletion *in vivo*.

Taken together, our results indicate that non-viral engineering of anti-CD117 CAR T cells can lead to potent antitumor activity and complete eradication of HSPCs, envisioning its application prior to alloHSCT in early clinical trials of patients with high-risk AML or MDS.

MATERIALS AND METHODS

Generation of CAR mRNA

The generation of the CD117 CAR from the 79D antibody,[21] including the CD8 hinge, CD8 transmembrane, 41BB costimulatory, and CD3zeta signaling domains was accomplished as previously described by us.[14] A PCR template encoding the mRNA CD117 CAR sequence under the control of a modified T7 promoter sequence was transcribed *in vitro* at the mRNA platform of the University Hospital of Zurich according to a previous report.[58] Capping and nucleotide modifications were introduced co-transcriptionally by having in the transcription reaction the CleanCap, Pseudouridine triphosphate and eventually (for the mRNA coding the CD117 CAR) 5-Methylcytidine triphosphate. When needed, the mRNA was polyadenylated after transcription using a recombinant poly-A polymerase as recommended by the manufacturer (NEB Biolabs).

Production of the LV encoding the CD117 CAR

The viral vector encoding the anti-CD117 CAR and the RQR8 sequences previously cloned in the pCDH-EF1α-MCS-T2A-GFP lentiviral plasmid (System Biosciences, Palo Alto, CA, USA) were produced as previously reported.[14] The RQR8 gene sequence was generously shared by Dr. Martin Pule (University College London, UK).[26] Briefly, HEK293T Lenti-X cells (Takara Bio Europe, Saint-Germain-en-Laye, France) were transfected with the CAR-encoding transfer vector, psPAX2 packaging, and pCAG-VSVG envelope plasmids (both kindly provided by Dr. Patrick Salmon, University of Geneva, Switzerland) using JetPRIME transfection reagent (Polyplus transfection, Illkirch, France). Viral particles were harvested 2 days later and concentrated with Peg-itTM (System Biosciences, Palo Alto, CA, USA).

Generation of SB iC9.CAR vector

The pT4.iC9.79D vector, which encodes the iC9 and the CD117 CAR sequences, was obtained from the pTMNDU3 plasmid[59] by replacing the CD19CAR and the inverted terminal repeats (ITRs). ITRs of the pT4 vector (Addgene, 117046)[23] and the iC9 gene[60] were synthesized by GeneArt (Life Technologies). The iC9 was inserted by restriction digestion into the LV under the control of a human EF1 promoter and in front of the CD117 CAR, separated by T2A element.[24] Functional analyses were performed on bulk cell products if not otherwise specified.

CAR T cell production with LV

anti-CD117 CAR T cells were produced by transduction of purified T cells from healthy donors as previously described.[14] Briefly, excess buffy coats of anonymized healthy blood donors were acquired from the Zurich blood donation service (Blutspende Zurich, Switzerland). Peripheral blood mononuclear cells (PBMCs) were isolated by density gradient centrifugation (Ficoll-Paque Plus, GE Healthcare, Chicago, Il, USA). T cells were negatively purified with EasySepTM beads (human T cell isolation kit, STEMCELL Technologies, Vancouver, Canada). T cells were cultured in Advanced RPMI 1640 enriched with FBS (10%), Glutamax, and Penicillin/Streptomycin (100 U/mL/100 μg/mL)

(Gibco, Thermo Fisher Scientific, Waltham, MA, USA) and stimulated with 80 U/mL IL-2 (Peprotech, Rocky Hill, NJ, USA). T cell activation was performed with CD3/CD28 Dynabeads (Thermo Fisher Scientific) followed by transduction with LV at MOI 3 in the presence of 8 μg/mL Hexadimethrine bromide (Sigma-Aldrich, St. Louis, MO, USA) the following day. On day 3, beads were removed with magnets. LV CAR T cells were cultured for 10 days before cryopreservation or immediate use.

CAR T cell production with mRNA

CAR T cells were produced by electroporation of purified T cells from healthy donors (Blutspende Zurich, Switzerland). T cell activation was performed with CD3/CD28 Dynabeads (Thermo Fisher Scientific) and 80 U/mL IL-2 (Peprotech, Rocky Hill, NJ, USA). mRNA anti-CD117 CARs were electroporated in T cells after 4 days post activation. Briefly, 5×10^6 T cells were resuspended in Human T cell Nucleofector Solution (Lonza) and electroporated in the presence of the anti-CD117 CAR mRNA using the Amaxa program T-20.

CAR T cell production with SB

SB CAR T cells were produced by adapting the previously reported method to conventional T cells.[61] Human T cells were purified with EasySepTM beads and resuspended in Human T cell Nucleofector Solution (Lonza). For each cuvette, 5×10^6 purified T cells were electroporated (program U-14) in presence of the pT4.iC9.79D vector and the transposase SB100X plasmid (Addgene 34879) or of the transposase SB100X mRNA. After electroporation, T cells were activated with CD3/CD28 Dynabeads (Thermo Fisher Scientific) and stimulated with 80 U/mL IL-2 (Peprotech, Rocky Hill, NJ, USA). CAR T cells were cultured for 10 days before cryopreservation or immediate use.

Cell lines

The HL60 and MOLM-14 cell lines were purchased from ATCC. The cell lines were cultured in RPMI 1640 supplemented with FBS (10%), and Penicillin/Streptomycin (100 U/mL/100 μg/mL) (Gibco, Thermo Fisher Scientific, Waltham, MA, USA). The HL-60 and MOLM-14 AML cell lines were modified to express a truncated version of the GNNK+ isoform of the *KIT* gene by LV gene transfer. The MOLM-14 AML cell line was co-transduced with the pCDH-EF1-Luciferase-T2A-GFP LV vector.[14] Transduced MOLM-14 cells were sorted according to the expression of CD117 and selected by limited dilution to generate cell lines expressing different levels of CD117 expression.

Flow cytometry and cell sorting

Cell suspension from *in vitro* culture or isolated from PB, BM, or spleen were stained with antibodies in 100 μL of phosphate-buffered saline supplemented with 2% FBS for 10 min at room temperature. Details on the antibodies used for immunophenotyping are reported in Table S1. Surface expression of the anti-CD117 CAR was detected with the Recombinant human c-Kit Protein (His Tag, SinoBiological), followed by the anti-CD117 antibody. In all analyses, the population was gated based on singlet gating followed by forward and side-scatter characteristics. All subsequent gates were set according to unstained controls. Live cells were gated using Zombie Aqua Fixable Viability Kit (Biolegend). For cell number quantification, Flow-Count Fluorospheres were used according to the manufacturer's instructions (Beckman Coulter, Pasadena, CA). Cells were acquired with an LSR II Fortessa cytometer (BD Bioscience, Franklin Lakes, NJ, USA) and the data were analyzed with FlowJo (version 10.0.8, LLC, Ashland, USA). To purify CAR+ T cells, CAR T cells were stained with CD3 and the Recombinant human c-Kit Protein, followed by anti-CD117 antibody and sorted with a FACS Aria III (BD Biosciences) or by CD117 Biotin (R&D) and sorted using Streptavidin Microbeads (Miltenyi).

Cytotoxic assay

Cytotoxicity was evaluated with a 24-hour co-culture assay as previously reported.[62] Cell death and apoptosis were detected using the GFP-Certified Apoptosis/Necrosis detection kit (Enzo Life Sciences, Farmingdale, NY), according to the manufacturer's instructions. T cells were co-cultured with targets at indicated E:T ratio. Target cells were identified according to GFP or were previously labeled with CFSE (1 μM, eBioscience). The final percentage of killed cells was determined as follows: (% of early necrotic and apoptotic co-cultured cells − % of early necrotic and apoptotic target cells alone) × 100/(100 − % of early necrotic and apoptotic target cells alone). All samples were run in duplicate. For long-term cytotoxic and proliferation assay, mRNA CAR T cells were stained with CellTrace CFSE Cell Proliferation Kit (Thermo Fisher Scientific) and co-cultured with target cells (E:T 1:1) as described previously.[63] After 3 days of incubation, cells were stained for expression of CAR, CD3, and CD117 (Table S1), and with Zombie Aqua Live/Dead (BioLegend, San Diego, CA, USA). Antibody staining followed by flow cytometry was used to evaluate target cell elimination and anti-CD117 CAR expression, while CAR T cell proliferation was determined by assessing CFSE staining dilution.

CID-mediated induction of apoptosis

T cells were cultured in the presence of AP20187 (2–200 nM). After 24 hours, cells were stained for expression of CAR, CD3, and with the Apoptosis/Necrosis detection kit (Enzo Life Sciences).

Apoptosis induction was assessed with flow cytometry. Survival was calculated by analyzing the percentage of viable residual cells in CD3+CAR+ cells considering the proportion of CAR+ cells, in the presence of AP20187 vs. medium alone as follows: (% of CAR+ alive cells) * (1 – (CAR+ cells with medium – CAR+ cells with AP20187)/(CAR+ cells with medium)).

Cytokine release assays

T cells/mL were stimulated with leukemic blasts at a ratio of 1:1. After 24 hours, culture supernatants were harvested and levels of INF-γ, TNF-α, and IL-2 were investigated with an enzyme-linked immunosorbent assay (ELISA MAX *Deluxe Set*, Biolegend, San Diego, CA, USA) according to the manufacturer's instruction. The samples were read with an absorbance plate reader (Tecan, D300 Digital Dispenser, Männedorf, ZH, Switzerland). The limit of detection was 7.8 pg/mL.

Primary human CB cells

CB-derived cells were isolated from placental tissue and processed by the biobank of the department of Medical Oncology and Hematology, University Hospital Zurich. Following the enrichment of mononucleated cells by density gradient centrifugation, CD34+ cells were purified by CD34 microbeads and LS columns (both Miltenyi Biotec). All primary human cells were cryo-preserved in freezing media. Human CB was obtained with parent written informed consent from cords/placentas of healthy full-term newborns (Department of Obstetrics, University Hospital Zurich and Triemli Hospital, Zurich).

Transgene copy number and western blot analysis

Average copy numbers of integrated SB transposons per genome in the polyclonal CAR T cell populations were determined by droplet digital PCR (ddPCR), as described in Prommersberger et al.[64] Briefly, genomic DNA samples prepared from the CAR T cells were subjected to digestion with DpnI to eliminate residual plasmid vector DNA, followed by fragmentation with CviQI. ddPCR reactions were performed by using two primer pairs for the target (the right terminal inverted repeat of the SB transposon) and the reference amplicons, as well as fluorescently labeled hydrolysis probes for both amplicons. Detection of fluorescence and counting of each droplet per ddPCR reaction was done with a droplet reader instrument (BioRad) using a two-color detection system. For western blot (WB) analysis, protein was harvested at 5 and 13 days post electroporation.[27,28]

In vivo studies

In vivo studies were conducted with NSG (NOD.Cg-Prkdc^{scid} Il2rg^{tm1Wjl}/SzJ) mouse strains. Animal experiments were conducted in compliance with procedures approved by the Veterinäramt des Kantons Zürich, Switzerland (194/2018 and 121/2022). The animals were kept in the animal facilities of the University Hospital of Zurich and the Schlieren Campus. Generation of humanized mice engrafted with human CB was performed as previously reported.[65] Briefly, newborn pups were sub-lethally irradiated with 150 cGy with an RS-2000 irradiator (Rad Source, Buford, GA, USA) and transplanted by intrahepatic injection of 1×10^5 CD34+ umbilical CB cells. Levels of chimerism were monitored by flow cytometry of PB post-transplantation. Administration of CAR T cells and NT cells was performed by intravenous injections 6–10 weeks post-transplantation. In the experiment depicted in Figure 1, mice received 2×10^6 mRNA CAR T cells, 6×10^6 mRNA CAR T cells, or 2×10^6 LV CAR T cells. Mice treated with 6×10^6 mRNA CAR T cells received an additional dose after 3 days. In the experiments depicted in Figure 5, mice received 2×10^6 CAR T cells. Animals were euthanized 6 days after T cell transfer in the experiment depicted in Figure 1 and 20 days after T cell transfer in the experiments described in Figure 5. BM puncture was performed to evaluate CD117+ cells before treatment with CID and ATG. For studies with CD117+, luciferase-GFP expressing MOLM-14 cells, adult NSG mice (6–9 weeks of age) were sub-lethally irradiated and intravenously injected with 0.1×10^6 MOLM-14 cells. For the experiment presented in Figure 4, animals with positive luciferase signal on day 9 were allocated to receive 2×10^6 CAR+ T cells, corresponding to 7.4×10^6 SB iC9.CAR T cells, 3.3×10^6 LV CAR T cells, 7.4×10^6 NT cells or PBS. For weekly bioluminescent imaging (BLI), mice were anesthetized and received 150 mg/kg bodyweight D-luciferin (PerkinElmer, Inc, Waltham, MA, USA) as intraperitoneal injection. Image acquisition was performed on a Xenogen IVIS 200 machine (PerkinElmer) with the Living Image 149 Software. For survival analysis, mice were closely monitored for any sign of suffering and promptly removed from the experiment in case of meeting humane endpoints. Hematological organs (PB, BM, and spleen) were collected and processed for flow cytometry analysis. For the experiment presented in Figure 6, animals with positive luciferase signal on day 11 were allocated to receive 1.4×10^6 purified CAR+ T cells in both the SB and LV conditions. Treatment of animals was performed with two subsequent daily doses of CID (50 μg/mouse, B/B Homodimerizer, AP20187, Takara Bio CA, USA), a single dose of Rituximab (500 μg/mouse), or three subsequent daily doses of ATG Rabbit (500 μg/mouse, Grafalon, Neovii, Germany).

Statistical analysis

Data are represented as mean ± standard error of the mean. One-way or two-way ANOVA with Turkey multiple comparisons were used to determine the statistical significance of the data. For survival analysis, statistical differences were calculated by Mantel-Cox test. Statistical analyses were performed with GraphPad Prism 5.0 (GraphPad Software, Inc., La Jolla, CA).

DATA AND CODE AVAILABILITY

Raw data that support the findings of this study that are not present in the manuscript will be made available upon request.

SUPPLEMENTAL INFORMATION

Supplemental information can be found online at https://doi.org/10.1016/j.omto.2023.07.003.

ACKNOWLEDGMENTS

This work was supported by the Swiss National Science Foundation (310030B_166673/1, to M.G.M. and PR00P3_201621 to C.F.M.), the Swiss Cancer Research (KFS-3846-02-2016), the Clinical Research Priority Program “ImmunoCure” of the University of Zurich to M.G.M. and D.N.; the Förderung des Akademischen Nachwuchses (FAN) of the University Hospital Zurich to C.F.M.; the University Research Priority Project Translational Cancer Research grant of the University of Zurich (to R.M., M.G.M., and S.P.). The authors would like to thank Norman Russkamp and Sandoval Villegas Nicolas for assistance in experiments, Andrea Biondi, and Giuseppe Gaipa for scientific discussion.

AUTHOR CONTRIBUTIONS

C.F.M. conceptualized the study, designed and performed all experiments, collected and analyzed the data, and wrote the manuscript. R.M. provided assistance in LV preparation and animal experiments with mRNA CAR T cells. S.B., M.C., M.P., L.V., F.M., and C.P. performed experiments and helped in preparing the figures. S.P. proposed the use of ivt mRNA for CAR and transposase, designed and

produced those mRNA molecules. Z.I. led the VCN and WB analysis, done by C.M. J.S. provided assistance in conceptualizing animal experiments. D.N. conceptualized the study and obtained financial support. M.G.M. conceptualized the study, designed the experiments, obtained financial support, supervised the study, and wrote and approved the manuscript. All authors reviewed and approved the final manuscript.

DECLARATION OF INTERESTS

The authors declare no competing interests.

REFERENCES

1. Horowitz, M., Schreiber, H., Elder, A., Heidenreich, O., Vormoor, J., Toffalori, C., Vago, L., and Kröger, N. (2018). Epidemiology and biology of relapse after stem cell transplantation. Bone Marrow Transpl. *53*, 1379–1389. https://doi.org/10.1038/s41409-018-0171-z.

2. Bonnet, D., and Dick, J.E. (1997). Human acute myeloid leukemia is organized as a hierarchy that originates from a primitive hematopoietic cell. Nat. Med. *3*, 730–737. https://doi.org/10.1038/nm0797-730.

3. Lapidot, T., Sirard, C., Vormoor, J., Murdoch, B., Hoang, T., Caceres-Cortes, J., Minden, M., Paterson, B., Caligiuri, M.A., and Dick, J.E. (1994). A cell initiating human acute myeloid leukaemia after transplantation into SCID mice. Nature *367*, 645–648. https://doi.org/10.1038/367645a0.

4. Thomas, D., and Majeti, R. (2017). Biology and relevance of human acute myeloid leukemia stem cells. Blood *129*, 1577–1585. https://doi.org/10.1182/blood-2016-10-696054.

5. Khaldoyanidi, S., Nagorsen, D., Stein, A., Ossenkoppele, G., and Subklewe, M. (2021). Immune Biology of Acute Myeloid Leukemia: Implications for Immunotherapy. J. Clin. Oncol. *39*, 419–432. https://doi.org/10.1200/jco.20.00475.

6. Finck, A.V., Blanchard, T., Roselle, C.P., Golinelli, G., and June, C.H. (2022). Engineered cellular immunotherapies in cancer and beyond. Nat. Med. *28*, 678–689. https://doi.org/10.1038/s41591-022-01765-8.

7. Majzner, R.G., and Mackall, C.L. (2019). Clinical lessons learned from the first leg of the CAR T cell journey. Nat. Med. *25*, 1341–1355. https://doi.org/10.1038/s41591-019-0564-6.

8. Tettamanti, S., Marin, V., Pizzitola, I., Magnani, C.F., Giordano Attianese, G.M.P., Cribioli, E., Maltese, F., Galimberti, S., Lopez, A.F., Biondi, A., et al. (2013). Targeting of acute myeloid leukaemia by cytokine-induced killer cells redirected with a novel CD123-specific chimeric antigen receptor. Br. J. Haematol. *161*, 389–401. https://doi.org/10.1111/bjh.12282.

9. Rotiroti, M.C., Buracchi, C., Arcangeli, S., Galimberti, S., Valsecchi, M.G., Perriello, V.M., Rasko, T., Alberti, G., Magnani, C.F., Cappuzzello, C., et al. (2020). Targeting CD33 in Chemoresistant AML Patient-Derived Xenografts by CAR-CIK Cells Modified with an Improved SB Transposon System. Mol. Ther. *28*, 1974–1986. https://doi.org/10.1016/j.ymthe.2020.05.021.

10. Tashiro, H., Sauer, T., Shum, T., Parikh, K., Mamonkin, M., Omer, B., Rouce, R.H., Lulla, P., Rooney, C.M., Gottschalk, S., and Brenner, M.K. (2017). Treatment of Acute Myeloid Leukemia with T Cells Expressing Chimeric Antigen Receptors Directed to C-type Lectin-like Molecule 1. Mol. Ther. *25*, 2202–2213. https://doi.org/10.1016/j.ymthe.2017.05.024.

11. Budde, L., Song, J.Y., Kim, Y., Blanchard, S., Wagner, J., Stein, A.S., Weng, L., Del Real, M., Hernandez, R., Marcucci, E., et al. (2017). Remissions of Acute Myeloid Leukemia and Blastic Plasmacytoid Dendritic Cell Neoplasm Following Treatment with CD123-Specific CAR T Cells: A First-in-Human Clinical Trial. Blood *130*, 811. https://doi.org/10.1182/blood.V130.Suppl_1.811.811.

12. Liu, F., Cao, Y., Pinz, K., Ma, Y., Wada, M., Chen, K., Ma, G., Shen, J., Tse, C.O., Su, Y., et al. (2018). First-in-Human CLL1-CD33 Compound CAR T Cell Therapy Induces Complete Remission in Patients with Refractory Acute Myeloid Leukemia: Update on Phase 1 Clinical Trial. Blood *132*, 901. https://doi.org/10.1182/blood-2018-99-110579.

13. Fiorenza, S., and Turtle, C.J. (2021). CAR-T Cell Therapy for Acute Myeloid Leukemia: Preclinical Rationale, Current Clinical Progress, and Barriers to Success. BioDrugs. *35*, 281–302. https://doi.org/10.1007/s40259-021-00477-8.

14. Myburgh, R., Kiefer, J.D., Russkamp, N.F., Magnani, C.F., Nuñez, N., Simonis, A., Pfister, S., Wilk, C.M., McHugh, D., Friemel, J., et al. (2020). Anti-human CD117 CAR T-cells efficiently eliminate healthy and malignant CD117-expressing hematopoietic cells. Leukemia *34*, 2688–2703. https://doi.org/10.1038/s41375-020-0818-9.

15. Ikeda, H., Kanakura, Y., Tamaki, T., Kuriu, A., Kitayama, H., Ishikawa, J., Kanayama, Y., Yonezawa, T., Tarui, S., and Griffin, J.D. (1991). Expression and functional role of the proto-oncogene c-kit in acute myeloblastic leukemia cells. Blood *78*, 2962–2968.

16. Escribano, L., Ocqueteau, M., Almeida, J., Orfao, A., and San Miguel, J.F. (1998). Expression of the c-kit (CD117) Molecule in Normal and Malignant Hematopoiesis. Leuk. Lymphoma *30*, 459–466. https://doi.org/10.3109/10428199809057558.

17. Kent, D., Copley, M., Benz, C., Dykstra, B., Bowie, M., and Eaves, C. (2008). Regulation of Hematopoietic Stem Cells by the Steel Factor/KIT Signaling Pathway. Clin. Cancer Res. *14*, 1926–1930. https://doi.org/10.1158/1078-0432.Ccr-07-5134.

18. Domen, J., and Weissman, I.L. (2000). Hematopoietic stem cells need two signals to prevent apoptosis; BCL-2 can provide one of these, Kitl/c-Kit signaling the other. J. Exp. Med. *192*, 1707–1718. https://doi.org/10.1084/jem.192.12.1707.

19. Russkamp, N.F., Myburgh, R., Kiefer, J.D., Neri, D., and Manz, M.G. (2021). Anti-CD117 immunotherapy to eliminate hematopoietic and leukemia stem cells. Exp. Hematol. *95*, 31–45. https://doi.org/10.1016/j.exphem.2021.01.003.

20. Magnani, C.F., Gaipa, G., Lussana, F., Belotti, D., Gritti, G., Napolitano, S., Matera, G., Cabiati, B., Buracchi, C., Borleri, G., et al. (2020). Sleeping Beauty-engineered CAR T cells achieve antileukemic activity without severe toxicities. J. Clin. Invest. *130*, 6021–6033. 1172/JCI138473.

21. Reshetnyak, A.V., Nelson, B., Shi, X., Boggon, T.J., Pavlenco, A., Mandel-Bausch, E.M., Tome, F., Suzuki, Y., Sidhu, S.S., Lax, I., and Schlessinger, J. (2013). Structural basis for KIT receptor tyrosine kinase inhibition by antibodies targeting the D4 membrane-proximal region. Proc. Natl. Acad. Sci. USA *110*, 17832–17837. https://doi.org/10.1073/pnas.1317118110.

22. Hoogenboom, H.R., and Winter, G. (1992). By-passing immunisation. Human antibodies from synthetic repertoires of germline VH gene segments rearranged in vitro. J. Mol. Biol. *227*, 381–388. https://doi.org/10.1016/0022-2836(92)90894-.

23. Wang, Y., Pryputniewicz-Dobrinska, D., Nagy, E.É., Kaufman, C.D., Singh, M., Yant, S., Wang, J., Dalda, A., Kay, M.A., Ivics, Z., and Izsvák, Z. (2017). Regulated complex assembly safeguards the fidelity of Sleeping Beauty transposition. Nucleic Acids Res. *45*, 311–326. https://doi.org/10.1093/nar/gkw1164.

24. Szymczak, A.L., Workman, C.J., Wang, Y., Vignali, K.M., Dilioglou, S., Vanin, E.F., and Vignali, D.A.A. (2004). Correction of multi-gene deficiency in vivo using a single 'self-cleaving' 2A peptide-based retroviral vector. Nat. Biotechnol. *22*, 589–594. https://doi.org/10.1038/nbt957.

25. Foster, M.C., Savoldo, B., Lau, W., Rubinos, C., Grover, N., Armistead, P., Coghill, J., Hagan, R.S., Morrison, K., Buchanan, F.B., et al. (2021). Utility of a safety switch to abrogate CD19.CAR T-cell-associated neurotoxicity. Blood *137*, 3306–3309. https://doi.org/10.1182/blood.2021010784.

26. Philip, B., Kokalaki, E., Mekkaoui, L., Thomas, S., Straathof, K., Flutter, B., Marin, V., Marafioti, T., Chakraverty, R., Linch, D., et al. (2014). A highly compact epitope-based marker/suicide gene for easier and safer T-cell therapy. Blood *124*, 1277–1287. https://doi.org/10.1182/blood-2014-01-545020.

27. Mátés, L., Chuah, M.K.L., Belay, E., Jerchow, B., Manoj, N., Acosta-Sanchez, A., Grzela, D.P., Schmitt, A., Becker, K., Matrai, J., et al. (2009). Molecular evolution of a novel hyperactive Sleeping Beauty transposase enables robust stable gene transfer in vertebrates. Nat. Genet. *41*, 753–761. https://doi.org/10.1038/ng.343.

28. Jin, Z., Maiti, S., Huls, H., Singh, H., Olivares, S., Mátés, L., Izsvák, Z., Ivics, Z., Lee, D.A., Champlin, R.E., and Cooper, L.J.N. (2011). The hyperactive Sleeping Beauty transposase SB100X improves the genetic modification of T cells to express a chimeric antigen receptor. Gene Ther. *18*, 849–856. https://doi.org/10.1038/gt.2011.40.

29. Kwon, H.S., Logan, A.C., Chhabra, A., Pang, W.W., Czechowicz, A., Tate, K., Le, A., Poyser, J., Hollis, R., Kelly, B.V., et al. (2019). Anti-human CD117 antibody-mediated

bone marrow niche clearance in nonhuman primates and humanized NSG mice. Blood *133*, 2104–2108. https://doi.org/10.1182/blood-2018-06-853879.

30. Eppert, K., Takenaka, K., Lechman, E.R., Waldron, L., Nilsson, B., van Galen, P., Metzeler, K.H., Poeppl, A., Ling, V., Beyene, J., et al. (2011). Stem cell gene expression programs influence clinical outcome in human leukemia. Nat. Med. *17*, 1086–1093. https://doi.org/10.1038/nm.2415.

31. Kim, M.Y., Yu, K.R., Kenderian, S.S., Ruella, M., Chen, S., Shin, T.H., Aljanahi, A.A., Schreeder, D., Klichinsky, M., Shestova, O., et al. (2018). Genetic Inactivation of CD33 in Hematopoietic Stem Cells to Enable CAR T Cell Immunotherapy for Acute Myeloid Leukemia. Cell *173*, 1439–1453.e19. https://doi.org/10.1016/j.cell.2018.05.013.

32. Ding, L., Saunders, T.L., Enikolopov, G., and Morrison, S.J. (2012). Endothelial and perivascular cells maintain haematopoietic stem cells. Nature *481*, 457–462. https://doi.org/10.1038/nature10783.

33. Perna, F., Berman, S.H., Soni, R.K., Mansilla-Soto, J., Eyquem, J., Hamieh, M., Hendrickson, R.C., Brennan, C.W., and Sadelain, M. (2017). Integrating Proteomics and Transcriptomics for Systematic Combinatorial Chimeric Antigen Receptor Therapy of AML. Cancer cell *32*, 506–519.e5. https://doi.org/10.1016/j.ccell.2017.09.004.

34. Wang, Q.S., Wang, Y., Lv, H.Y., Han, Q.W., Fan, H., Guo, B., Wang, L.L., and Han, W.D. (2015). Treatment of CD33-directed chimeric antigen receptor-modified T cells in one patient with relapsed and refractory acute myeloid leukemia. Mol. Ther. *23*, 184–191. https://doi.org/10.1038/mt.2014.164.

35. Zhang, H., Wang, P., Li, Z., He, Y., Gan, W., and Jiang, H. (2021). Anti-CLL1 Chimeric Antigen Receptor T-Cell Therapy in Children with Relapsed/Refractory Acute Myeloid Leukemia. Clin. Cancer Res. *27*, 3549–3555. https://doi.org/10.1158/1078-0432.CCR-20-4543.

36. Jin, X., Zhang, M., Sun, R., Lyu, H., Xiao, X., Zhang, X., Li, F., Xie, D., Xiong, X., Wang, J., et al. (2022). First-in-human phase I study of CLL-1 CAR-T cells in adults with relapsed/refractory acute myeloid leukemia. J. Hematol. Oncol. *15*, 88. https://doi.org/10.1186/s13045-022-01308-1.

37. Sauer, T., Parikh, K., Sharma, S., Omer, B., Sedloev, D., Chen, Q., Angenendt, L., Schliemann, C., Schmitt, M., Müller-Tidow, C., et al. (2021). CD70-specific CAR T cells have potent activity against acute myeloid leukemia without HSC toxicity. Blood *138*, 318–330. https://doi.org/10.1182/blood.2020008221.

38. Leick, M.B., Silva, H., Scarfò, I., Larson, R., Choi, B.D., Bouffard, A.A., Gallagher, K., Schmidts, A., Bailey, S.R., Kann, M.C., et al. (2022). Non-cleavable hinge enhances avidity and expansion of CAR-T cells for acute myeloid leukemia. Cancer cell *40*, 494–508.e5. https://doi.org/10.1016/j.ccell.2022.04.001.

39. Moretti, A., Ponzo, M., Nicolette, C.A., Tcherepanova, I.Y., Biondi, A., and Magnani, C.F. (2022). The Past, Present, and Future of Non-Viral CAR T Cells. Front. Immunol. *13*, 867013. https://doi.org/10.3389/fimmu.2022.867013.

40. Chaudhary, N., Weissman, D., and Whitehead, K.A. (2021). mRNA vaccines for infectious diseases: principles, delivery and clinical translation. Nat. Rev. Drug Discov. *20*, 817–838. https://doi.org/10.1038/s41573-021-00283-5.

41. Pascolo, S. (2021). Synthetic Messenger RNA-Based Vaccines: from Scorn to Hype. Viruses *13*. https://doi.org/10.3390/v13020270.

42. Henderson, J.M., Ujita, A., Hill, E., Yousif-Rosales, S., Smith, C., Ko, N., McReynolds, T., Cabral, C.R., Escamilla-Powers, J.R., and Houston, M.E. (2021). Cap 1 Messenger RNA Synthesis with Co-transcriptional CleanCap(®) Analog by In Vitro Transcription. Curr. Protoc. *1*, e39. https://doi.org/10.1002/cpz1.39.

43. Karikó, K., Muramatsu, H., Welsh, F.A., Ludwig, J., Kato, H., Akira, S., and Weissman, D. (2008). Incorporation of pseudouridine into mRNA yields superior nonimmunogenic vector with increased translational capacity and biological stability. Mol. Ther. *16*, 1833–1840. https://doi.org/10.1038/mt.2008.200.

44. Foster, J.B., Choudhari, N., Perazzelli, J., Storm, J., Hofmann, T.J., Jain, P., Storm, P.B., Pardi, N., Weissman, D., Waanders, A.J., et al. (2019). Purification of mRNA Encoding Chimeric Antigen Receptor Is Critical for Generation of a Robust T-Cell Response. Hum. Gene Ther. *30*, 168–178. https://doi.org/10.1089/hum.2018.145.

45. Beatty, G.L., Haas, A.R., Maus, M.V., Torigian, D.A., Soulen, M.C., Plesa, G., Chew, A., Zhao, Y., Levine, B.L., Albelda, S.M., et al. (2014). Mesothelin-specific chimeric antigen receptor mRNA-engineered T cells induce anti-tumor activity in solid malignancies. Cancer Immunol. Res. *2*, 112–120. https://doi.org/10.1158/2326-6066.CIR-13-0170.

46. Rurik, J.G., Tombácz, I., Yadegari, A., Méndez Fernández, P.O., Shewale, S.V., Li, L., Kimura, T., Soliman, O.Y., Papp, T.E., Tam, Y.K., et al. (2022). CAR T cells produced in vivo to treat cardiac injury. Science *375*, 91–96. https://doi.org/10.1126/science.abm0594.

47. Cummins, K.D., Frey, N., Nelson, A.M., Schmidt, A., Luger, S., Isaacs, R.E., Lacey, S.F., Hexner, E., Melenhorst, J.J., June, C.H., et al. (2017). Treating Relapsed/Refractory (RR) AML with Biodegradable Anti-CD123 CAR Modified T Cells. Blood *130*, 1359. https://doi.org/10.1182/blood.V130.Suppl_1.1359.1359.

48. Ito, T., Smrž, D., Jung, M.Y., Bandara, G., Desai, A., Smržová, Š., Kuehn, H.S., Beaven, M.A., Metcalfe, D.D., and Gilfillan, A.M. (2012). Stem cell factor programs the mast cell activation phenotype. J. Immunol. *188*, 5428–5437. https://doi.org/10.4049/jimmunol.1103366.

49. Di Stasi, A., Tey, S.K., Dotti, G., Fujita, Y., Kennedy-Nasser, A., Martinez, C., Straathof, K., Liu, E., Durett, A.G., Grilley, B., et al. (2011). Inducible apoptosis as a safety switch for adoptive cell therapy. N. Engl. J. Med. *365*, 1673–1683. https://doi.org/10.1056/NEJMoa1106152.

50. Arai, Y., Choi, U., Corsino, C.I., Koontz, S.M., Tajima, M., Sweeney, C.L., Black, M.A., Feldman, S.A., Dinauer, M.C., and Malech, H.L. (2018). Myeloid Conditioning with c-kit-Targeted CAR-T Cells Enables Donor Stem Cell Engraftment. Mol. Ther. *26*, 1181–1197. https://doi.org/10.1016/j.ymthe.2018.03.003.

51. Ivics, Z. (2021). Potent CAR-T cells engineered with Sleeping Beauty transposon vectors display a central memory phenotype. Gene Ther. *28*, 3–5. https://doi.org/10.1038/s41434-020-0138-8.

52. Gattinoni, L., Lugli, E., Ji, Y., Pos, Z., Paulos, C.M., Quigley, M.F., Almeida, J.R., Gostick, E., Yu, Z., Carpenito, C., et al. (2011). A human memory T cell subset with stem cell-like properties. Nat. Med. *17*, 1290–1297. https://doi.org/10.1038/nm.2446.

53. Fraietta, J.A., Lacey, S.F., Orlando, E.J., Pruteanu-Malinici, I., Gohil, M., Lundh, S., Boesteanu, A.C., Wang, Y., O'Connor, R.S., Hwang, W.T., et al. (2018). Determinants of response and resistance to CD19 chimeric antigen receptor (CAR) T cell therapy of chronic lymphocytic leukemia. Nat. Med. *24*, 563–571. https://doi.org/10.1038/s41591-018-0010-1.

54. Czechowicz, A., Kraft, D., Weissman, I.L., and Bhattacharya, D. (2007). Efficient transplantation via antibody-based clearance of hematopoietic stem cell niches. Science *318*, 1296–1299. https://doi.org/10.1126/science.1149726.

55. Agarwal, R., Dvorak, C.C., Prockop, S., Kwon, H.-S., Long-Boyle, J.R., Le, A., Brown, J.W., Merkel, E., Truong, K., Velasco, B., et al. (2021). JSP191 As a Single-Agent Conditioning Regimen Results in Successful Engraftment, Donor Myeloid Chimerism, and Production of Donor Derived Naïve Lymphocytes in Patients with Severe Combined Immunodeficiency (SCID). Blood *138*, 554. https://doi.org/10.1182/blood-2021-153074.

56. Chhabra, A., Ring, A.M., Weiskopf, K., Schnorr, P.J., Gordon, S., Le, A.C., Kwon, H.S., Ring, N.G., Volkmer, J., Ho, P.Y., et al. (2016). Hematopoietic stem cell transplantation in immunocompetent hosts without radiation or chemotherapy. Sci. Transl. Med. *8*, 351ra105. https://doi.org/10.1126/scitranslmed.aae0501.

57. Wunderlich, M., Brooks, R.A., Panchal, R., Rhyasen, G.W., Danet-Desnoyers, G., and Mulloy, J.C. (2014). OKT3 prevents xenogeneic GVHD and allows reliable xenograft initiation from unfractionated human hematopoietic tissues. Blood *123*, e134–e144. https://doi.org/10.1182/blood-2014-02-556340.

58. Tusup, M., French, L.E., De Matos, M., Gatfield, D., Kundig, T., and Pascolo, S. (2019). Design of in vitro Transcribed mRNA Vectors for Research and Therapy. Chimia (Aarau) *73*, 391–394. https://doi.org/10.2533/chimia.2019.391.

59. Magnani, C.F., Mezzanotte, C., Cappuzzello, C., Bardini, M., Tettamanti, S., Fazio, G., Cooper, L.J.N., Dastoli, G., Cazzaniga, G., Biondi, A., and Biagi, E. (2018). Preclinical Efficacy and Safety of CD19CAR Cytokine-Induced Killer Cells Transfected with Sleeping Beauty Transposon for the Treatment of Acute Lymphoblastic Leukemia. Hum. Gene Ther. *29*, 602–613. https://doi.org/10.1089/hum.2017.207.

60. Straathof, K.C., Pulè, M.A., Yotnda, P., Dotti, G., Vanin, E.F., Brenner, M.K., Heslop, H.E., Spencer, D.M., and Rooney, C.M. (2005). An inducible caspase 9 safety switch for T-cell therapy. Blood *105*, 4247–4254. https://doi.org/10.1182/blood-2004-11-4564.

61. Magnani, C.F., Turazzi, N., Benedicenti, F., Calabria, A., Tenderini, E., Tettamanti, S., Giordano Attianese, G.M.P., Cooper, L.J.N., Aiuti, A., Montini, E., et al. (2016). Immunotherapy of acute leukemia by chimeric antigen receptor-modified lymphocytes using an improved Sleeping Beauty transposon platform. Oncotarget *7*, 51581–51597.

62. Turazzi, N., Fazio, G., Rossi, V., Rolink, A., Cazzaniga, G., Biondi, A., Magnani, C.F., and Biagi, E. (2018). Engineered T cells towards TNFRSF13C (BAFFR): a novel strategy to efficiently target B-cell acute lymphoblastic leukaemia. Br. J. Haematol. *182*, 939–943. https://doi.org/10.1111/bjh.14899.

63. Gagliani, N., Magnani, C.F., Huber, S., Gianolini, M.E., Pala, M., Licona-Limon, P., Guo, B., Herbert, D.R., Bulfone, A., Trentini, F., et al. (2013). Coexpression of CD49b and LAG-3 identifies human and mouse T regulatory type 1 cells. Nat. Med. *19*, 739–746. https://doi.org/10.1038/nm.3179.

64. Prommersberger, S., Monjezi, R., Botezatu, L., Miskey, C., Amberger, M., Mestermann, K., Hudecek, M., and Ivics, Z. (2022). Generation of CAR-T Cells with Sleeping Beauty Transposon Gene Transfer. Methods Mol. Biol. *2521*, 41–66. https://doi.org/10.1007/978-1-0716-2441-8_3.

65. Saito, Y., Ellegast, J.M., Rafiei, A., Song, Y., Kull, D., Heikenwalder, M., Rongvaux, A., Halene, S., Flavell, R.A., and Manz, M.G. (2016). Peripheral blood CD34(+) cells efficiently engraft human cytokine knock-in mice. Blood *128*, 1829–1833. https://doi.org/10.1182/blood-2015-10-676452.

OMTO, Volume *30*

Supplemental information

Anti-CD117 CAR T cells incorporating a safety switch eradicate human acute myeloid leukemia and hematopoietic stem cells

Chiara F. Magnani, Renier Myburgh, Silvan Brunn, Morgane Chambovey, Marianna Ponzo, Laura Volta, Francesco Manfredi, Christian Pellegrino, Steve Pascolo, Csaba Miskey, Zoltán Ivics, Judith A. Shizuru, Dario Neri, and Markus G. Manz

Supplemental Information

Table S1. List of fluorescently labeled antibodies/proteins used for flow cytometry.

Target	Clone	Fluorochrome	Supplier
Protein-A	n/a	FITC	Thermo Fisher
CD3	OKT3	APC	BioLegend
CD4	RPA-T4	FITC	Invitrogen
CD8	SK1	PerCP-Cy5.5	BioLegend
CD45RA	HI100	Brilliant Violet 711™	BioLegend
CD62L	DREG-56	PB	BioLegend
CD117	104D2	PE-Cy7	Thermo Fisher
CD27	M-T271	BV510	Biolegend
CD95	DX2	BV786	BD Biosciences
CD279	EH12.2H7	APC Cy7	Biolegend
CD223	3DS223H	Alexa-Fluor 700	BD Biosciences
CD366	F38-2E2	PE	Ebiosciences
mouse CD45	30-F11	PerCP-Cy5.5	BioLegend
CD34	QBend-10	PE	Thermo Fisher
CD33	WM53	Brilliant Violet 711™	BioLegend
CD19	SJ21C1	FITC	BioLegend
CD45	HI30	eFluor™ 450	Thermo Fisher
HLA-A2	BB7.2	APC-780	BD Biosciences

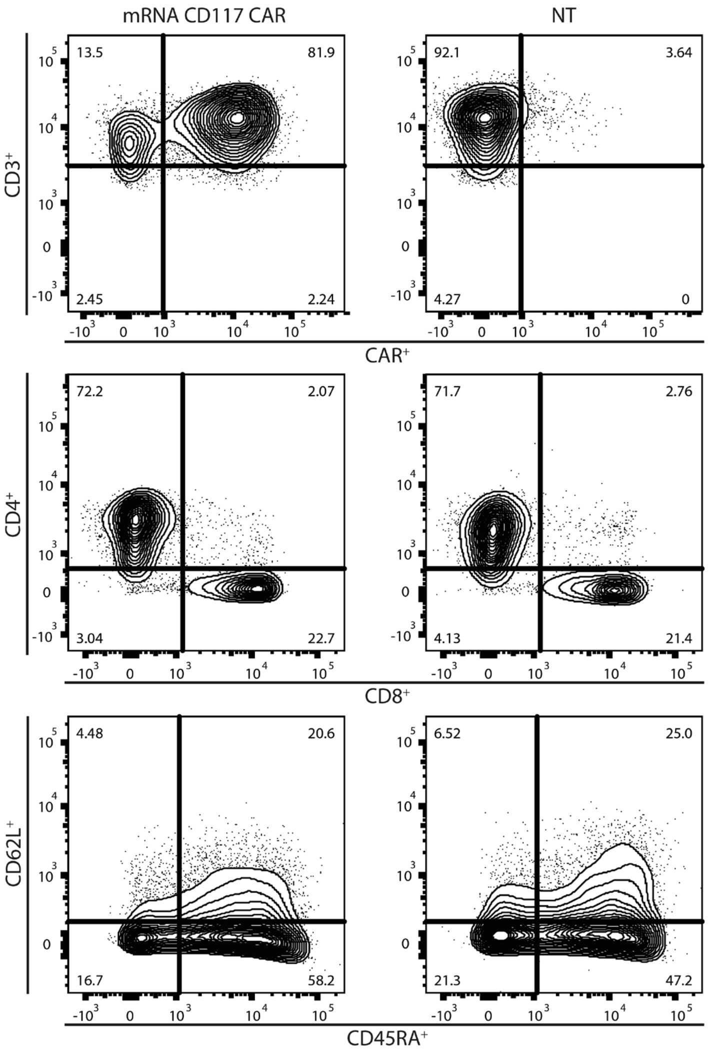

Figure S1: Phenotype of T cells electroporated with mRNA CD117CAR.
Purified T cells were stimulated and electroporated with 10 µg of CAR mRNA or in absence of DNA (not transfected, NT). Flow cytometric immunophenotyping by dual density plots in one representative donor (n = 5). CD3/CAR, CD4/CD8, CD45RA/CD62L expression were measures.

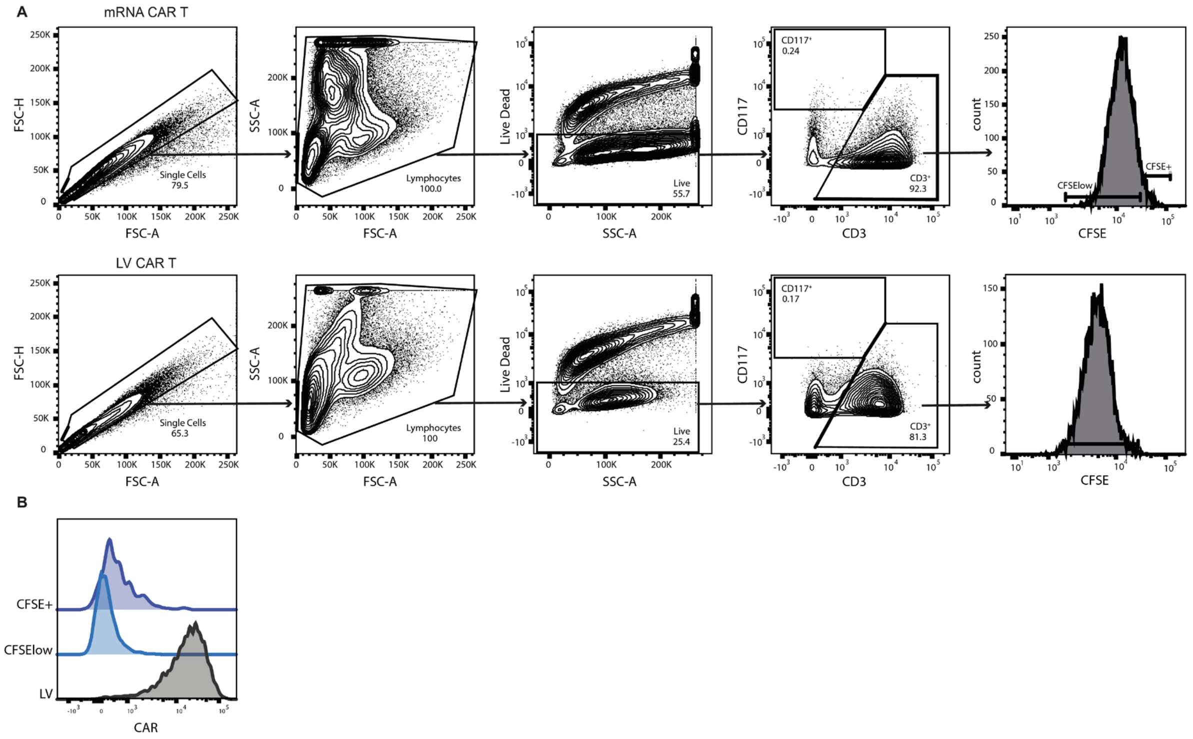

Figure S2: Ivt mRNA CD117 CAR T cells demonstrate efficient killing in vitro.
(A) CFSE stained T cells were co-culture with CD117+ HL-60 target cells for three days. Representative flow cytometric immunophenotyping showing the gating strategy in one representative donor (n = 3). Analyses were performed by gating into alive cells before evaluating lineage markers (CD117 and CD3) to retrieve target and effector cell percentages. (B) CAR expression of T cells gated according to CFSE retention in mRNA CAR T cells compared to LV CAR T cells. One representative histogram of results from three donors is shown.

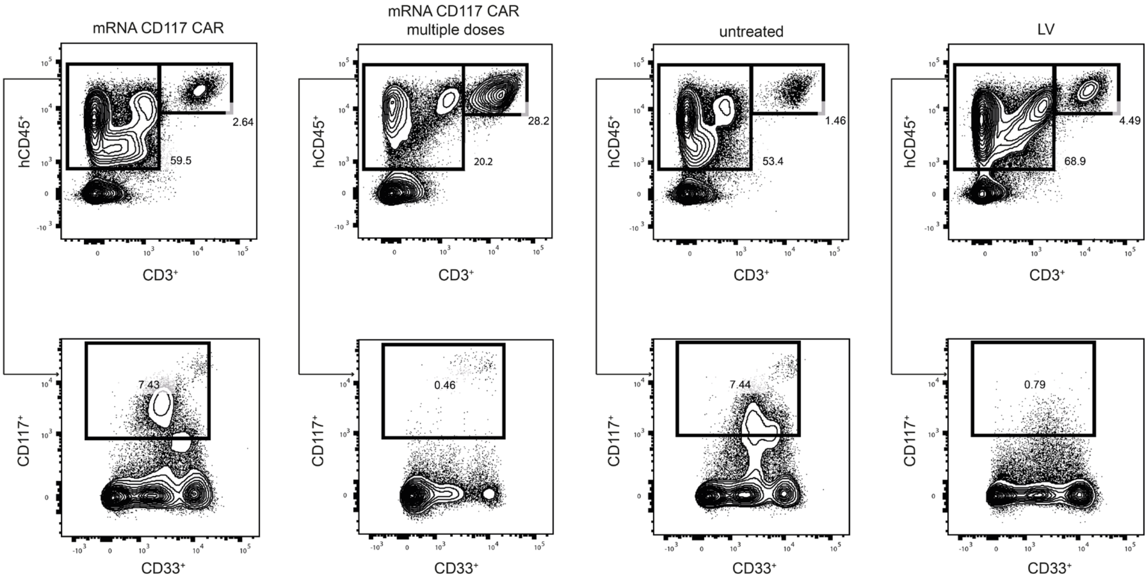

Figure S3: mRNA CD117 CAR T cells deplete healthy CD117 HSPCs in vivo.
Newborn NSG mice were sublethally irradiated and injected with CB-derived hCD34+ cells. After having confirmed the engraftment and establishment of human hematopoiesis, mice received a single dose of mRNA CAR T cells (2 x 10^6), two high doses mRNA CAR T cells (6 x 10^6 every three days), or a single dose of LV CAR T cells (2 x 10^6). Representative Flow cytometric immunophenotyping of the bone marrow of treated animals at endpoint. One representative mouse is shown (n=3).

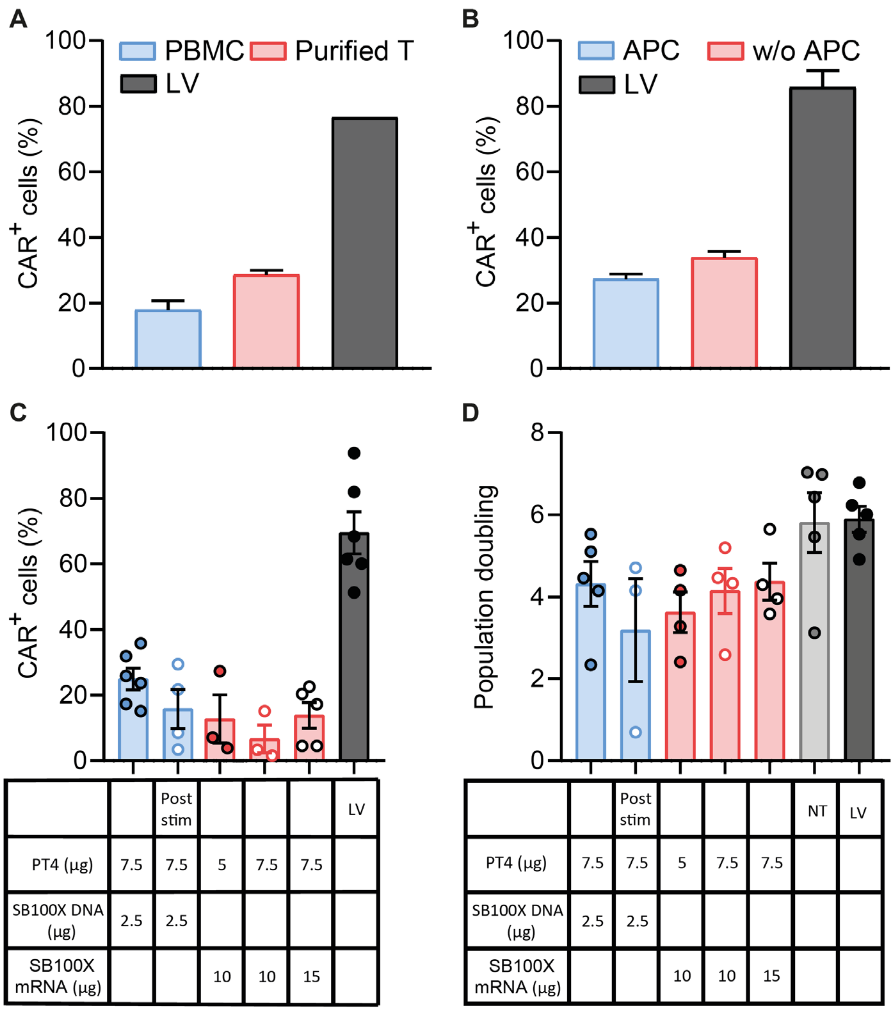

Figure S4: Transduction optimization of CD117 CAR T cells by SB vector.
(A) CAR T cells were generated by electroporation of total PBMCs or purified T cells in the presence of SB vector and the SB100X, compared to LV transduced purified T cells. CAR expression as percentage of CD3 cells as determined by flow cytometry with the recombinant c-Kit protein at 10 days after electroporation. (B) CAR expression as percentage of CD3 cells in T cells electroporated with SB vectors and stimulated in the presence or absence of APC, compared to LV transduced T cells. (C) CAR expression as percentage of CD3 cells of T cells electroporated with different concentrations of the PT4 vector and SB100X transposase, provided as DNA plasmid or mRNA, before or post stimulation, compared to LV transduced T cells, analyzed by flow cytometry at 10 days after electroporation. (D) Population doubling of T cells electroporated with different concentrations of the PT4 vector and SB100X transposase, provided as DNA plasmid or mRNA, before or post stimulation, compared to LV transduced T cells, analyzed by flow cytometry at 10 days after electroporation.

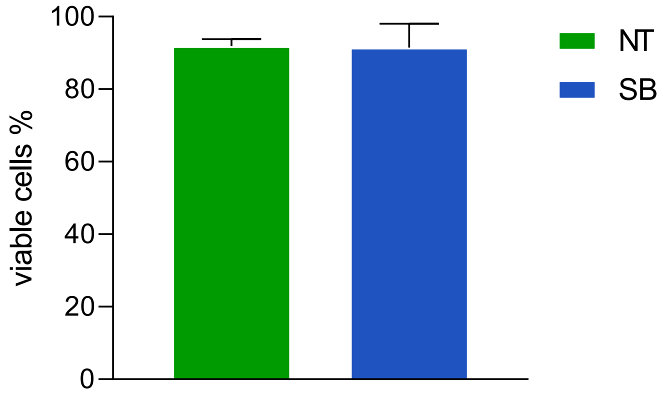

Figure S5: Viability of CD117 CAR T cells generated by the SB vector at 24 hours after electroporation. CAR T cells were generated by electroporation of purified T cells in the presence of the SB vector and the SB100X DNA and cell viability was assessed at 24 hours after electroporation. Data illustrate the mean ±SD from two different donors.

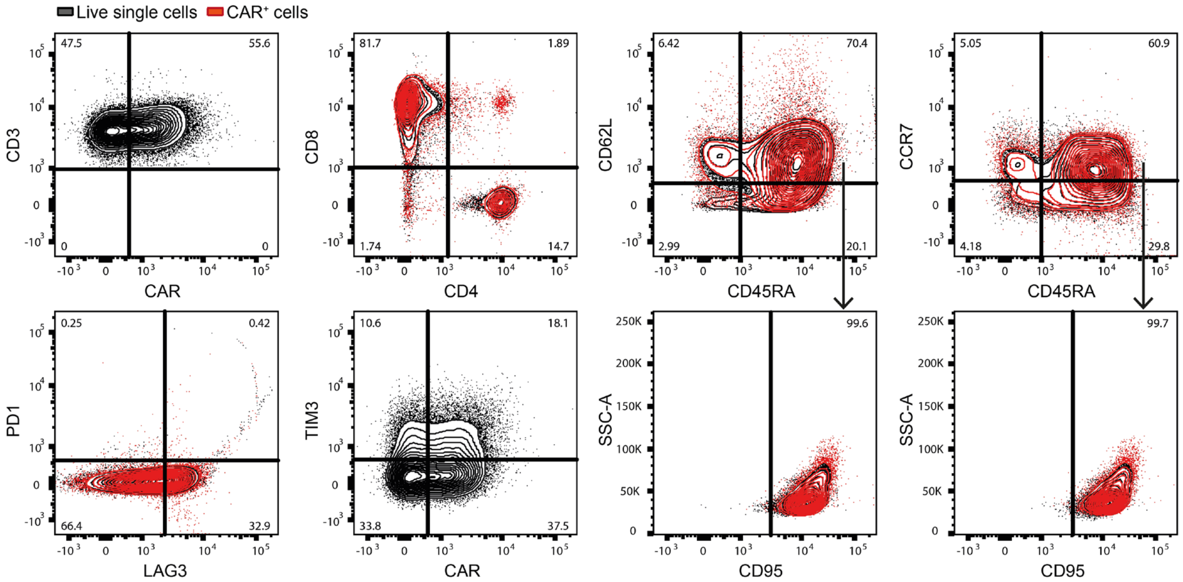

Figure S6: Phenotypical characterization of CD117 CAR T cells generated by SB vector.
CAR T cells were generated by electroporation of purified T cells in the presence of the SB vector and the SB100X. At the end of the 10-day differentiation protocol, cells were stained and acquired by flow cytometry. Dual-density plots in one representative batch are shown.

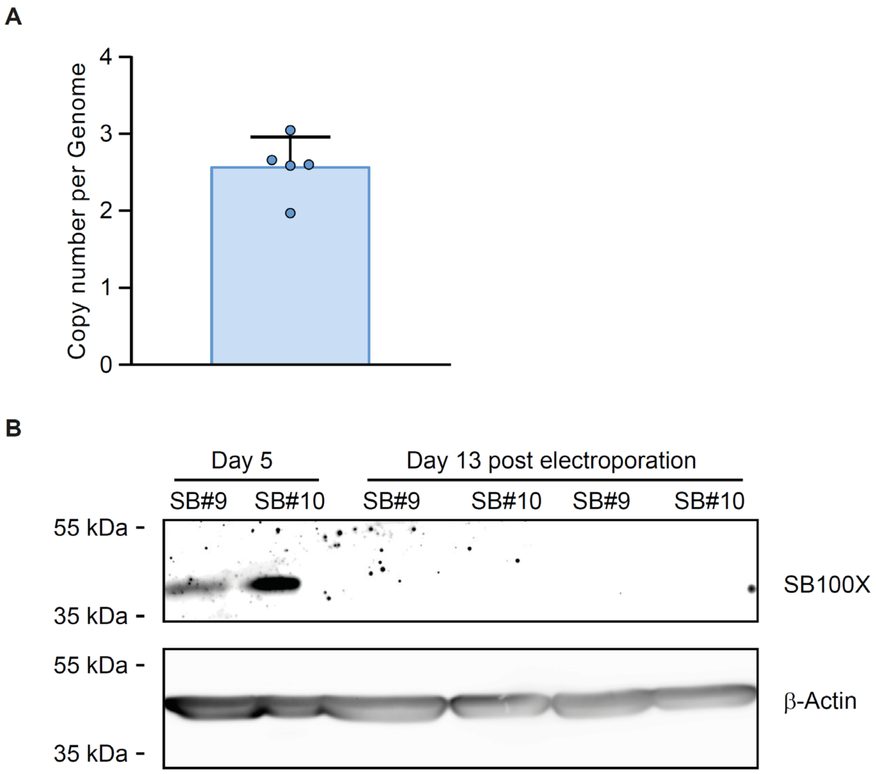

Figure S7: Transgene copy number and residual SB100X protein in CD117 CAR T cells generated by SB vector.
CAR T cells were generated by electroporation of total PBMC or purified T cells in the presence of SB vector and the SB100X. (A) Transgene copy number per genome as determined by digital PCR at 10 days after electroporation. (B) SB100X protein evaluated by western blot using protein extract isolated from NT and SB iC9.CAR T cells (SB100X DNA) at 5 and 13 days post electroporation. Data illustrate protein extract from 2 different donors, donor #9 and donor #10.

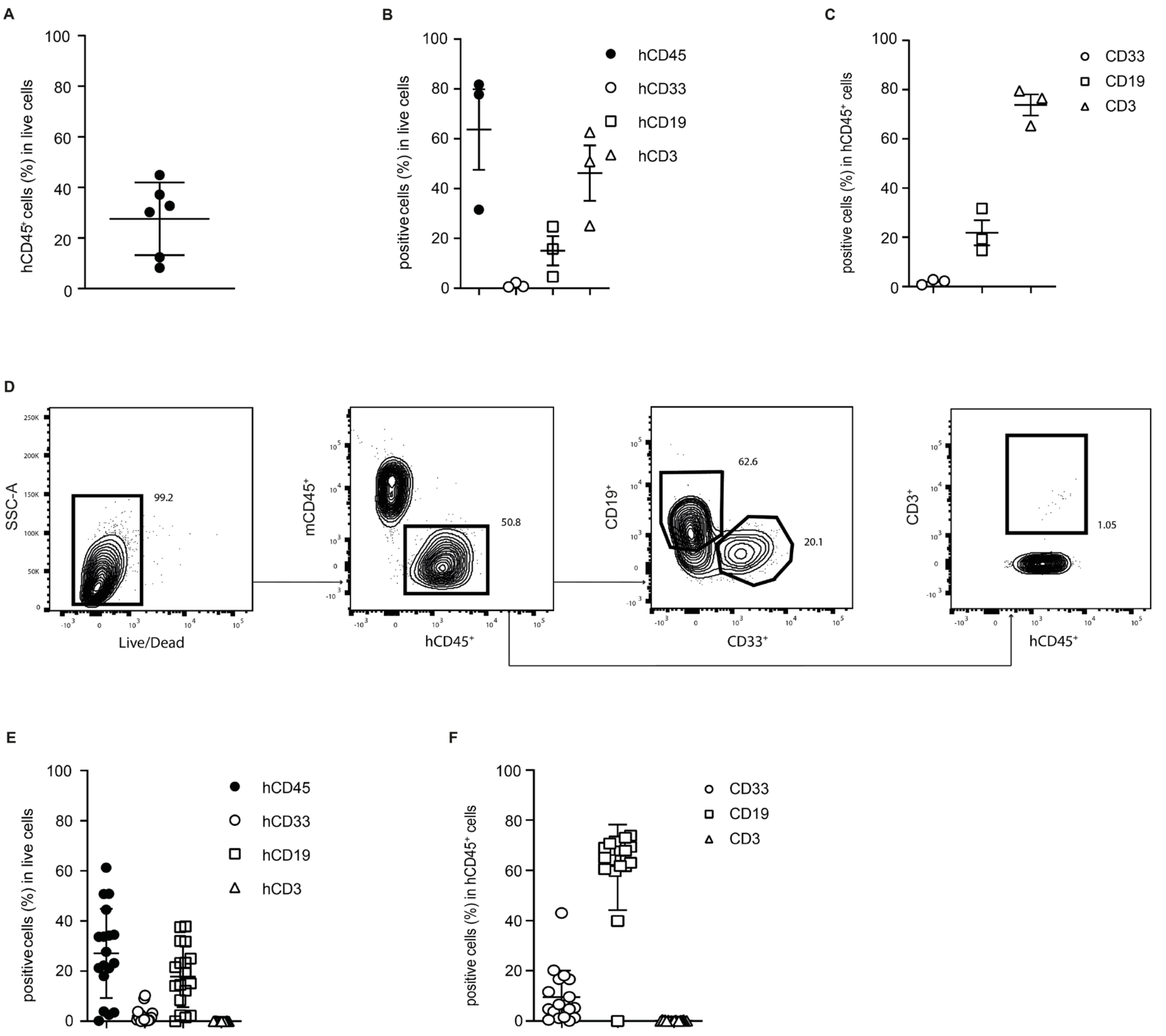

Figure S8: SB-engineered CD117 CAR T cells deplete healthy CD117 HSPCs in vivo.
(A) Human engraftment as percentage of hCD45 cells in live cells from peripheral blood of humanized mice, analyzed by flow cytometry at 40 days after transplantation. (B) Human engraftment as percentage of hCD45, hCD33, hCD19, and hCD3 cells in live cells from peripheral blood of humanized mice, analyzed by flow cytometry two weeks after CAR T-cell treatment (54 days after transplantation). (C) Human engraftment as percentage of hCD33, hCD19, and hCD3 cells in hCD45 cells from peripheral blood of humanized mice, analyzed by flow cytometry two weeks after CAR T cell treatment (54 days after transplantation). (D) Representative flow cytometric immunophenotyping with the gating strategy of the PB of mice engrafted with human CD34+ CB cells. One representative mouse is shown. (E) Human engraftment as percentage of hCD45, hCD33, hCD19, and hCD3 cells in live cells from peripheral blood of humanized mice, analyzed by flow cytometry at 60 days after transplantation. (F) Human engraftment as percentage of hCD33, hCD19, and hCD3 cells in hCD45 cells from peripheral blood of humanized mice, analyzed by flow cytometry at 60 days after transplantation.
